# Potential of phytochemicals in the treatment of Alzheimer disease by modulating lysosomal dysfunction: a systematic review

**DOI:** 10.1186/s13020-025-01204-z

**Published:** 2025-09-01

**Authors:** Man Yuan, Trinh Thach Thi Nguyen, Alasdair J. Gibb, Yan-Fang Xian, Hong-Xi Xu

**Affiliations:** 1 School of Pharmacy, Shanghai University of Traditional Chinese Medicine, No. 1200 Cailun Road, Shanghai, 201203 China; 2 Engineering Research Center of Shanghai Colleges for T.C.M. New Drug Discovery, Shanghai, 201203 China; 3 Department of Neuroscience, Physiology and Pharmacology, University College London, London, UK; 4 School of Chinese Medicine, Faculty of Medicine, The Chinese University of Hong Kong, Shatin, N.T., Hong Kong SAR, China

**Keywords:** Alzheimer disease, Lysosomes, Phytochemicals, Autophagy, Phagocytosis

## Abstract

Alzheimer disease (AD) is a primary international health dilemma, especially in elderly populations, due to its progressive nature and its adverse cognitive impact. The dysfunction of lysosomes, which impairs protein degradation and leads to toxic accumulation in neurons, is a pivotal factor in AD. We explore phytochemicals that specifically target lysosomal dysfunction via the activation of autophagy, phagocytosis, and lysosome function, exhibiting anti-inflammatory and antioxidant properties. This study involves extracting and evaluating phytochemicals by exploring multiple databases, Google Scholar, PubMed, the Science Citation Index Expanded (SCIE), and the China National Knowledge Infrastructure (CNKI), integrating contemporary biochemical evidence with TCM principles—highlighting the interconnected roles of deficiency, stasis, and phlegm—to provide a comprehensive therapeutic framework. Key phytochemicals—magnolol, trehalose, and salidroside- demonstrate notable promise in enhancing lysosomal function, reducing amyloid beta accumulation, and improving cognitive outcomes. Addressing traditional theory and modern science, we underline the potential for future research by clarifying the mechanisms of compounds and their effectiveness, which may delay the disease process.

**Graphical Abstract:**

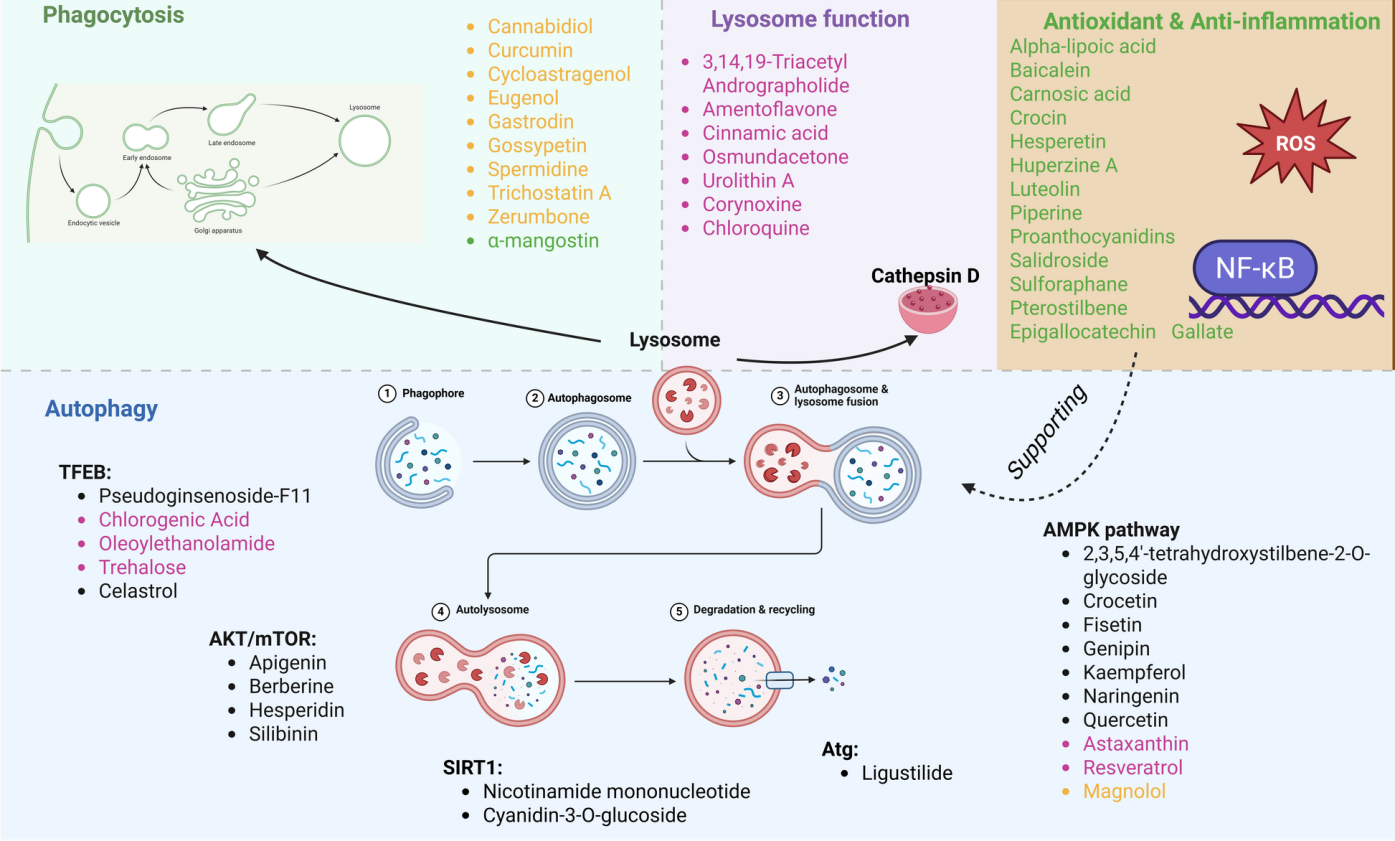

**Supplementary Information:**

The online version contains supplementary material available at 10.1186/s13020-025-01204-z.

## Introduction

Alzheimer disease (AD) is the predominant dementia etiology, identified as the fifth leading cause of death worldwide [1, 2], impacting an estimated 45 million people globally [1]. In 2050, the rate of AD is projected to increase almost twice in Europe and triple globally [2], the main risk element for AD include senior age, generally more than 65 [3, 4], and women, particularly those over 80, face a greater chance of developing AD in comparison with men [2].

Currently, approved medications for AD are designated for the stage of clinical dementia, focus on modulating neurochemical systems to address cognitive impairments and behavioural symptoms, and offer only temporary symptomatic relief [1, 5]. Over the past 25 years, translational studies have supported a hypothetical model describing AD pathophysiology leading to an early build-up of amyloid beta (Aβ) species and plaques inside the brain up to 20–30 years before the subsequent expansion of tau, neuronal loss, and the eventual onset of clinical symptoms [1, 6].

In light of the limited efficacy of current symptomatic treatments, recent research has shifted toward disease-modifying strategies targeting upstream mechanisms of AD pathology. Lysosomal function has garnered growing interest due to its central role in degrading misfolded proteins, particularly Aβ and tau [7]. Modern experimental therapies aim to enhance autophagy-lysosomal flux or restore lysosomal acidification, showing promise in preclinical models [8, 9]. However, the interventions remain in early-stage development and often face poor bioavailability or systemic toxicity, the potential of phytochemicals with multi-target capabilities, as promising candidates for modulating lysosomal pathways in a safer and more integrative manner.

Recent evidence increasingly suggests a connection between autophagy-lysosomal, endocytic–lysosomal pathway dysfunction and the progression of AD [10, 11]. Lysosomes serve an essential function in AD by their involvement in the degradation and clearing of Aβ and tau aggregates [12].

While the mechanistic insights are rooted in modern neurobiology, they resonate with traditional Chinese medicine (TCM) theories, which describe AD as a condition driven by the interconnected factors of deficiency, stasis, and phlegm. Deficiency—chiefly Kidney weakness and Spleen Qi decline—starves neural tissue, downregulates lysosomal activity, and slows turnover of other organelles, so Aβ and tau accumulate. Stasis, expressed as blood stasis and qi stagnation in cerebral collaterals, restricts microcirculation, heightens oxidative stress, and blocks glymphatic flow, retaining Aβ in parenchyma and amplifying tissue hypoxia. Phlegm, generated when Spleen transport falters and Kidney Yang weakens, fosters endogenous turbid deposits that seed plaque formation, drive amyloid aggregation, activate microglia, and overload the lysosome–autophagy axis [13–18]. Three processes lock into a feed-forward loop: deficiency weakens cellular waste clearance, stasis traps neurotoxic metabolites, and phlegm ignites low-grade neuroinflammation; each element intensifies the other two, accelerating synaptic loss and neuronal death [18]. The convergence of modern mechanisms and traditional theories provides a rational basis for identifying phytochemicals that simultaneously target TCM syndromes and molecular pathologies [15].

Natural products demonstrate exceptional efficacy in enhancing cerebral circulation, restoring injured nerve tissue, and improving the function of impaired nerves [19, 20]. By modulating critical cellular pathways, phytochemicals improve lysosomal activity, exhibit anti-inflammatory and antioxidant properties, and promote autophagy and protein degradation, reducing the buildup of Aβ and tau, thus providing a multifaceted strategy for AD therapy [20, 21]. This review aims to systematically summarize recent advances in phytochemicals that modulate lysosomal dysfunction in Alzheimer disease and clarify their mechanisms of action. We hypothesize that targeting lysosomal impairment through phytochemicals provides a multi-target, integrative therapeutic strategy that may overcome the limitations of current single-target drugs.

### Search strategy and inclusion criteria

**Fig. 1:**
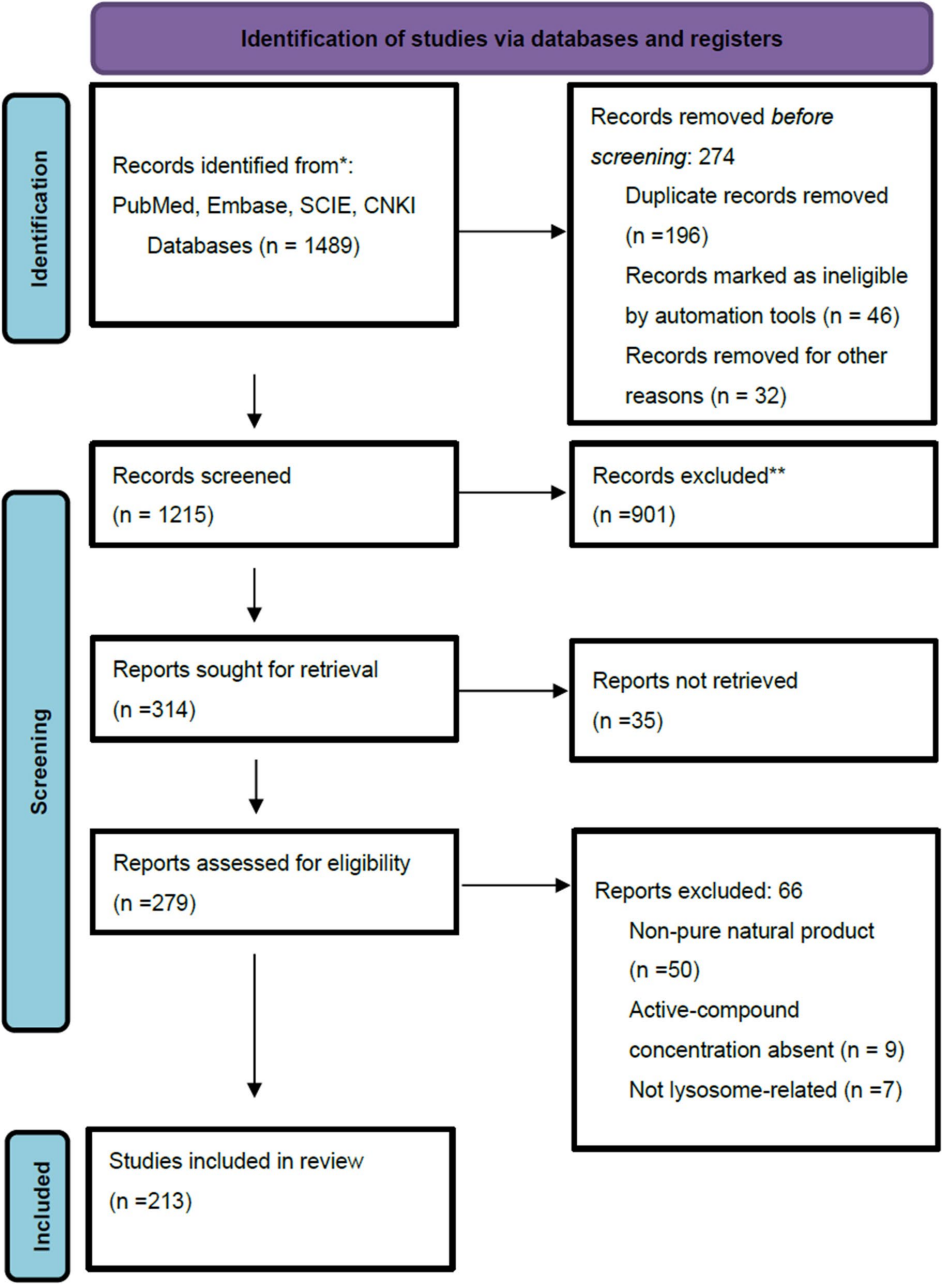
Prisma Flow Chart

A systematic review search was performed across multiple electronic databases, comprising Google Scholar, PubMed, the Science Citation Index Expanded (SCIE), and the China National Knowledge Infrastructure (CNKI). The search terms used were “Alzheimer disease,” “Alzheimer Dementia,” “lysosomal dysfunction,” “autophagy,” “phagocytosis,” “natural compound,” and “phytochemicals.” Only studies published in English from 2015 to the present were included. Studies were eligible if they explored the influence of phytochemicals on lysosomal function, autophagy, phagocytosis or neurodegenerative processes in AD and were published in peer-reviewed journals; the inclusion criteria encompassed selecting only compounds with demonstrated efficacy in ≥ 2 independent studies, mechanistic evidence across multiple AD models (in vitro + in vivo), and dose-dependent effects on lysosomal pathways. Studies were excluded if they focused on conditions other than AD, did not address lysosomal dysfunction, were not peer-reviewed, were conference abstracts, were based on animal models with insufficient data, or were issued in non-English languages – PRISMA Flow chart (Fig. 1).

### Mechanisms of lysosomal dysfunction in AD and phytochemical modulators

#### Pathological drivers of lysosomal dysfunction in AD

Lysosomal dysfunction plays a pivotal role in the pathogenesis of AD, closely associated with pathological processes (Aβ plaque accumulation, neurofibrillary tangles, chronic neuroinflammation, and oxidative stress), which converge to impair the lysosomal-autophagy system, establishing a vicious cycle that accelerates neurodegeneration [12, 22, 23].

**Fig. 2:**
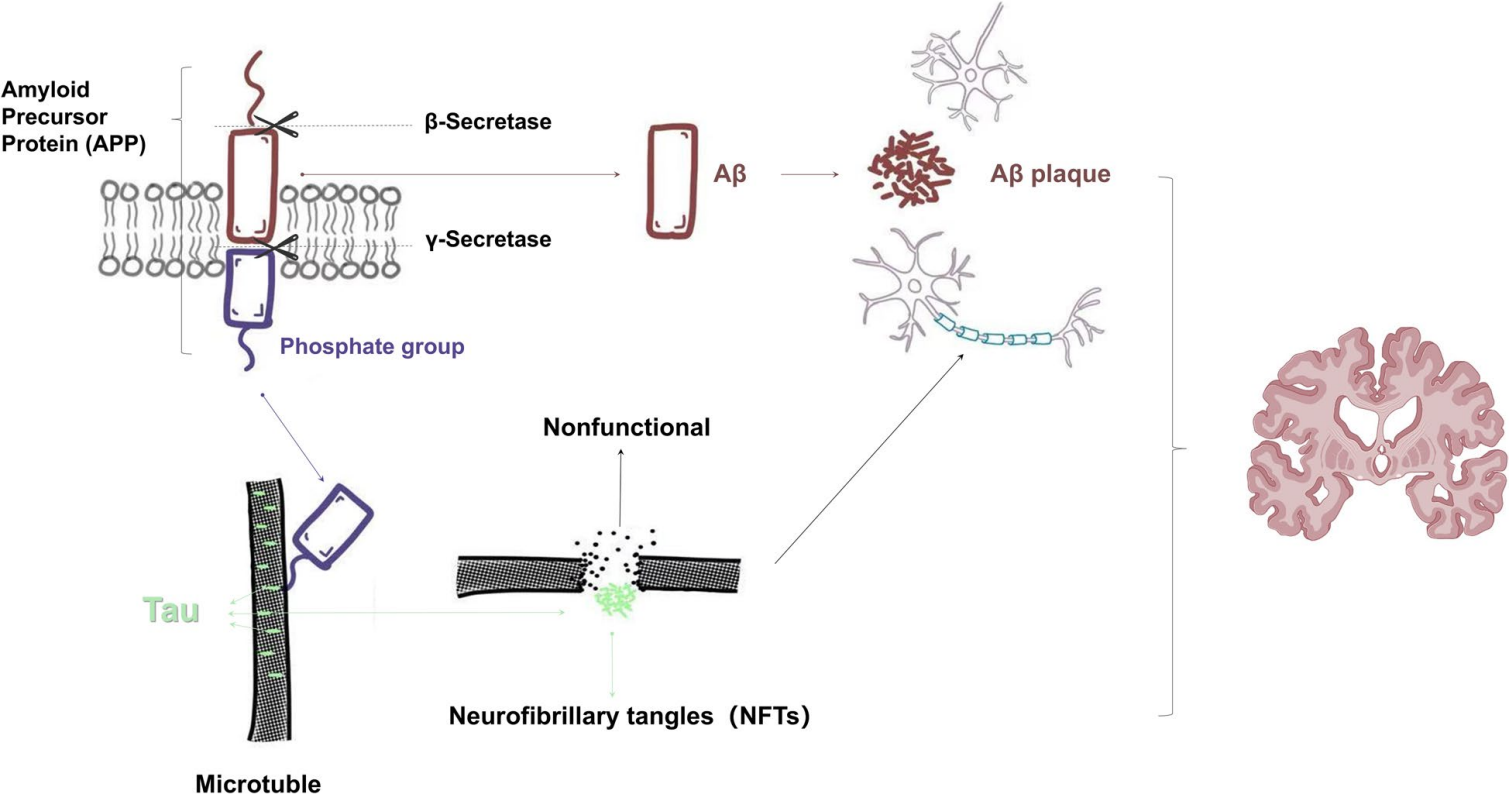
Pathological processes in Alzheimer Disease

In AD, Aβ peptides, derived from amyloid precursor protein (APP), aggregate into neurotoxic oligomers, particularly Aβ42, which self-aggregate into extracellular plaques [1, 3, 24–27] (Fig. 2), which disrupt synapses and activate microglia, which attempt to clear them but often fail [28, 29]. Aβ also promotes tau hyperphosphorylation via kinases—CDK5 and GSK3β, leading to neurofibrillary tangle formation and further toxicity [30]. Recent findings show that tau phosphorylation, especially at biomarker sites, renders tau resistant to degradation by lysosomal proteases and correlates with lysosomal protease levels in cerebrospinal fluid [31]. Excessive phosphorylation of Aβ and tau binds to the subunits of v-ATPase, leading to a decline in endolysosomal activity [32].

The lysosomes, an acidic compartment containing many hydrolases, contain about 60 enzymes that help to break down various biological molecules [23, 33–37], working most effectively at an acidic pH range of 4.5 to 5 [36]. Enzymes like cathepsins B, D, and L break down damaged proteins, and v-ATPases maintain acidity by pumping protons into the lysosomal lumen [35, 38, 39]. In AD, impaired v-ATPase activity raises lysosomal pH, suppressing cathepsin function and reducing Aβ clearance [1, 38, 40–42].

Neuroinflammation further exacerbates lysosomal impairment, activated microglia and astrocytes release interleukin-1β (IL-1β) and IL-6, forming a sustained inflammatory environment [43, 44]. Microglia also show impaired phagocytic capacity and prolonged activation, while astrocytes hinder Aβ clearance and modulate microglial phenotype via IL-3 [43, 45–52]. Meanwhile, oxidative stress generates reactive aldehydes like 4-hydroxynonenal and malondialdehyde, destabilizing lysosomal membranes, disrupting pH homeostasis, and inhibiting cathepsin activity [53–55]. Mitochondrial dysfunction exacerbates this process, compromising ATP-dependent lysosomal acidification and fusion [53, 56].

The accumulation of pathological proteins, chronic inflammation, and oxidative damage mutually reinforce each other, driving lysosomal failure [57–59]. Some phytochemicals—magnolol, berberine, and ginsenosides have demonstrated potential to restore lysosomal acidification, enhance autophagic clearance, and modulate cathepsin activity, providing promising avenues for alleviating AD-related lysosomal dysfunction.

### Phytochemicals from traditional Chinese medicine targeting lysosomal dysfunction in AD

Recent pharmacological studies have identified numerous bioactive compounds derived from traditional Chinese medicinal (TCM) herbs that exhibit therapeutic potential for AD by targeting lysosomal dysfunction [60–62]. The phytochemicals often act on multiple targets, including restoration of lysosomal acidification, enhancement of autophagic flux, regulation of cathepsin activity, and promotion of Aβ clearance. Baicalein from *Scutellaria baicalensis* (Huang Qin), naringenin from *Citrus reticulata* (Chen Pi), ginsenosides from *Panax ginseng* (Ren Shen), ligustilide from *Angelica sinensis* (Dang Gui), and magnolol from *Magnolia officinalis* (Hou Po) have demonstrated antioxidant, anti-inflammatory, and lysosome-enhancing activities in various in vitro and in vivo AD models [63–67], underscoring the value of TCM-derived phytochemicals as modulators of lysosomal function in AD, highlighting their promise as multitarget agents in developing novel therapeutics.

#### Phytochemical modulation of microglial phagocytosis to enhance lysosomal clearance in AD

In AD, microglial phagocytosis serves as a key lysosome-dependent mechanism for the clearance of Aβ and maintaining neuronal homeostasis [68, 69]. Phagocytosis involves internalizing extracellular cargo through receptor-mediated or receptor-independent pathways as a specialized form of endocytosis [70]. The accumulation of Aβ in the brain elicits a localized immune response that recruits microglia and astrocytes to sites of plaque formation, where they attempt to phagocytose aggregated Aβ [68]. However, progressive Aβ deposition disrupts endocytic trafficking and impairs phagocytic signaling, particularly in astrocytes, leading to reduced clearance capacity for Aβ oligomers and exacerbating plaque burden [70].

Caveolin-mediated and clathrin-mediated endocytosis are critical routes for Aβ internalization [71], in caveolin-mediated endocytosis, caveolae enhance the capture of extracellular Aβ peptides, bypassing lysosomal degradation of the delivered cargo, thereby increasing the efficiency of the intracellular cargo transport mechanism [72]. Increased expression of caveolin-1 potentially reduces Aβ; caveolin-1 is also diminished in AD patients; it is affected by elevated blood glucose levels, which disrupt amyloid metabolism and increase tau phosphorylation [71].

In clathrin-mediated endocytosis (CME), the main route for APP incorporation into clathrin-coated vesicles at the plasma membrane, and PICALM, a Phosphatidylinositol-binding clathrin assembly key protein, reducing PICALM expression diminishes APP internalization and lowers Aβ production [71], and abnormal cleavage of PICALM is also associated with neurofibrillary tangles, co-localizing with abnormally structured tau and increased tau phosphorylation (p-tau), contributing to AD intracellular dysfunction [71, 73]. CD36 (Cluster of Differentiation 36) mediates the innate host response to Aβ; when Aβ binds to the receptors triggers cellular reactions [74, 75]. Clathrin, adaptins, and dynamin are involved in the endocytosis process and prepare it for subsequent transport to lysosomes; it is triggered by clathrin interaction with the Adaptor protein complex 2, which binds clathrin to the plasma membrane and recognizes specific cargo molecules [76].

Additionally, Aβ accumulation alters receptor expression, triggering a receptor expressed on myeloid cells 2, disrupting microglial activation and phagocytic efficiency [71, 77]. Metabolic changes—specifically increased glycolysis and reduced mitochondrial oxidative phosphorylation—impair microglial phagocytosis, resulting in poor Aβ clearance and accidental removal of healthy synapses, which worsens neurodegeneration. Disturbances can influence the pathological phagocytosis process in metabolic pathways and dysregulation of "find-me" and "eat-me" signaling on neurons [68]. Activated PPARγ (peroxisome proliferator-activated receptor gamma) translocates to the microglial nucleus, upregulating Mertk, a key phagocytic receptor. Elevated Mertk enhances microglia’s recognition of phosphatidylserine “eat-me” signals on myelin debris or apoptotic cells, resulting in more efficient phagocytosis [78]. The study in 2021 by Alexandra Grubman also highlighted that the TREM2 receptor plays a vital role in the phagocytic process [77, 79]. Furthermore, the study demonstrated that XO4 + microglia (using labelling with methoxy-XO4, XO4 +) have a more substantial capacity for endocytosis of Aβ and synaptic proteins, which impact neuroinflammation and synapse loss in AD [79].

Phytochemicals have been extensively studied for their potential in alleviating AD through enhancing phagocytosis, particularly lysosome-mediated mechanisms in microglial cells – curcumin and cyanidin-3-O-glucoside have shown significant promise, as shown in Table 1. Some compounds act directly on key molecular targets involved in phagocytosis, potently enhancing microglial Aβ clearance. Curcumin modulates innate immune gene expression, particularly increasing TREM2 while decreasing CD33 expression, thus enhancing microglial phagocytosis and reducing pro-inflammatory phenotypes [80]. Cyanidin-3-O-glucoside regulates microglial polarization from M1 to M2 via PPARγ activation, increasing phagocytic activity through TREM2 modulation [81, 82].

**Table 1:** Pharmacologically active compounds in targeting phagocytosis

Group	Compounds	Structure	Species	Activities	Mechanism	Models	Dose	Behavioral experiment results	Ref
Flavonoid	Amentoflavone		*Ginkgo biloba* – Yin Xing	Promotes the cellular uptake of Aβ peptides (Aβ1-40 and Aβ1-42), mediated by endocytosis, inhibiting lysosomal enzymes, and protects cells from Aβ-induced cytotoxicity	Via class A scavenger receptors, indicating a selective transport mechanism, inhibiting lysosomal enzymes (with leupeptin), increased the accumulation of Aβ inside the cells	N2a BV2	1 µM, 3 µM, and 10 µM (3–24 h)		[163]
Curcuminoid	Curcumin		*Curcuma longa –* Jiang Huang	Promotes autophagy, destabilization of lysosomal membranes, rebalances innate immune gene expression, reduces pro-inflammatory markers, enhances microglial migration and phagocytosis of amyloid plaques, and reduces miR-155	Accumulates in the lysosome and alters the permeability of the lysosomal membrane, increases ROS, stimulates the formation of autophagic vacuoles, upregulates TREM2 and TyroBP/DAP12, promoting tyrosine kinase signaling and microglial activation for phagocytosis, downregulates CD33, an inhibitory receptor that opposes TREM2 function, enabling improved plaque clearance, increases expression of CD68 and Arg1, decreases expression of CD11b, iNOS, COX-2, and C1q	Huh-7 THP-1 BV2 cell Tg2576 (APPSwe) transgenic apoE3–5xFAD mice ged wild-type C57Bl6/J mice	5 μM to 25 μM (24–48 h) 0.1 μM-1.5 μM (24–72 h) 160 ppm- 500 ppm (5- 6 months)		[80, 164]
Flavonoid (Anthocyanin)	Cyanidin-3-O-glucoside		*Chrysanthemum* spp – Ju Hua	Reduces the Aβ40 and Aβ42, APP, PSEN1, and BACE1 in the hippocampus and cortex, increases autophagy flux, and has anti-inflammatory and antioxidant effects	Activates AMPK → SIRT1 and up-regulates PI3K/Akt, p-GSK3β, suppresses MAPKs, via APP/BACE1/PS1 down-regulation Downregulates (IL-1β, IL-6, TNF-α), ROS generation, microglial shift from M1 (CD86, CD80) to M2 (CD206, CD163), increases Aβ42 phagocytosis, increases PPARγ and TREM2 expression	HMC3 cells APP/PS1 mice APPswe/PS1ΔE9 transgenic mice	1 µM 30 mg/kg/day (16 weeks) 30 mg/kg/day (38 weeks)	Improved spatial working memory (increased spontaneous alternation percentage with no change in total arm entries)	[81, 82]
Triterpene aglycone	Cycloastragenol		*Astragalus membranaceus* – Huang Qi	Lowers hippocampal Aβ plaque burden and boosts whole-brain glucose metabolism, decreases the number of senescent microglia, and enhances microglial phagocytosis of Aβ	Binds and inhibits phosphodiesterase-4B**,** raises cAMP and activates p-CREB/BDNF signalling, rejuvenates microglia by clearing senescent cells, restoring phagocytic competence, and curbing neuroinflammation	BV2 5 × FAD × CX3CR1	1–10 nmol/L 25 or 75 mg/kg/day (3 months)	Enhanced spatial learning and reference memory (Morris Water Maze escape latencies across training days 1–7 shifted left, and probe-day time and platform crossings in the target quadrant rose), with unchanged swim speed and cue-trial performance ruling out motor or visual confounds	[88]
Flavonoid	Epigallocatechin Gallate		*Camellia sinensis* L. Ktze. (Theaceae) – Lv Cha	Inhibits the phagocytosis of Aβ by microglia, anti-inflammation	Reduces microglial phagocytic activity without relying on metal chelation	Primary microglia from 1- to 3-day-old male CD1 mice	1 µM		[165]
Phenylpropanoid	Eugenol		*Syzygium aromaticum* – Ding Xiang	Reduces Aβ plaque load and neuronal degeneration in cortex & hippocampus, suppresses neuroinflammation	Inhibits necroptosis (decreases pMLKL/MLKL), protecting neurons, dampens microglia/astrocyte activation and pro-inflammatory cytokines, promotes M2-like microglial polarization and up-regulates MACRO, CD36, CD68 – enhancing Aβ phagocytosis and lysosomal clearance, lowers Aβ without altering APP, PS1, IDE, or NEP levels	Transgenic 5 × FAD mice	10—30 mg/ kg/day (2 months)	Enhanced working memory (Y-maze spontaneous-alternation percentage rebounded to wild-type levels) and spatial learning plus reference memory (Morris Water Maze training escape latencies shortened; probe-day crossings, time, and swim distance in the target quadrant increased), with unchanged swim speed ruling out motor confounds	[83]
Phenolic glycoside	Gastrodin		*Gastrodia elata* – Tian Ma	Reduces neuroinflammation, inhibits phagocytosis, suppresses microglial activation	Involves the TLR4/TRAF6/NF-κB pathway and enhancing Stat3 phosphorylation, suppressing microglial activation, and shifting microglia from M1 to M2 phenotype	BV-2 cell C57BL/6 mice	100 mg/kg/day (5 days) 1 μg/ml and 10 μg/ml	Enhancing spatial learning and memory (reduced escape latency)	[84]
Flavonoid	Gossypetin		*Hibiscus sabdariffa* – Luo Shen Kui	Neuroprotective, lowers brain Aβ load (plaques, oligomers, monomers), antioxidant	Boosts microglial Aβ clearance, up-regulates phagocytosis genes (*Lpl*, *Clec7a), increases phagosome formation and microglial MHC-II⁺ fraction, speeds Aβ uptake, down-regulates pro-inflammatory DAM markers (*Apoe*, *Spp1*), reducing DAM signature and gliosis	Primary mouse microglia & BV2 microglial cell line 5xFAD transgenic mice	25 µM, (24 h) 10 mg/ kg (13 weeks)	Enhanced working memory, exploration, and spatial learning and reference memory (Y-maze spontaneous-alternation percentages rebounded toward wild-type with unchanged arm entries, while Morris Water Maze training escape latencies shifted left and probe-day time—and crossings—in the target quadrant rose, reflecting quicker acquisition and stronger recall	[87]
	Oleoylethanolamide			Increases expression of genes in lipid homeostasis, regulators of lysosomal function, microglial phagocytosis, and suppression of neuroinflammation	Activation of PPARα via its stable analog KDS-5104 increased the expression of genes involved in lipid homeostasis, including CYP4A, enhanced TFEB, enhances lysosomal biogenesis through a mTORC1-independent pathway, increases the microglial uptake of Aβ plaques dependent on PPARα and is mediated by the receptor CD36, reduced lipid droplet accumulation in microglia, and reduced the inflammatory response induced by LPS	Primary microglial cultures, BV2 cells, and HeLa cells 5xFAD mice	10 mg/kg (2 months)	Cognitive Improvement (increased exploration of novel objects and improved associative learning)	[62]
Polyamine	Spermidine		*Spinacia oleracea* – Bo Cai	Decreases soluble Aβ and pro-inflammatory cytokines, enhances Aβ phagocytosis & microglial motility	Increases Beclin-1, ETS2; broad rise in autophagy-related protein, up-regulation of AXL → GAS6 axis & actin-nucleation gene ARPC3 boosts Aβ clearance, suppresses NF-κB phosphorylation, lowering IL-6, TNF-α, and Il-1β transcription, interferes with NLRP3-inflammasome assembly, cutting IL-1β / IL-18 release	Primary neonatal & adult microglia; primary astrocytes APPPS1 transgenic mice	3 – 10 µM (15–18 h) 3 mM (290 days)		[166, 167]
Hydroxamic acid	Trichostatin A		*Streptomyces hygroscopicus*	Markedly reduces hippocampal Aβ-plaque number & area and lowers soluble Aβ and Aβ-oligomer levels in both brain & plasma, attenuates Abnormal microglial proliferation	Class-I HDAC inhibition, up-regulation of albumin in microglia & endothelial cells; albumin binds Aβ, blocks its fibrillisation, recruits microglia, and ferries Aβ across blood vessels to the periphery, enhances microglial phagocytosis/endothelial endocytosis of Aβ oligomers; fosters intracellular Aβ removal via the ubiquitin–proteasome pathway (UPP) rather than autophagy	BV2 microglia, bEnd.3 endothelial, HT22 neurons, N2a neuroblastoma APP/PS1	60–250 nM (optimal 125 nM 2 mg/kg/day (30 days)	Enhanced recognition memory and spatial learning/reference memory (Novel Object Recognition index after a 6-h delay rebounded to wild-type levels, while Morris Water Maze escape latencies on training days 4–5 shortened and probe-day time—and crossings—in the target quadrant increased), with unchanged swim speed and visual-cue performance ruling out motor or visual confounds	[86]
Sesquiterpenoid	Zerumbone		*Zingiber zerumbet* Smith – Shan Jiang	Reduces neuroinflammation, shifts microglial phenotype from M1 to M2, enhances microglial Aβ phagocytosis, and reduces synaptic loss in the hippocampus	Inhibits MAPK/NF-κB signaling, reduces phosphorylation of MAPK, ERK1/2, and p65, suppresses PGE2, COX-2, and mPGES-1 production. Promotes M2 microglia (increases CD206 and ARG-1 expression)—blocks NF-κB nuclear translocation	N9 microglial cell Primary microglia from C57BL/6 mice Transgenic APP/PS1 mice Wild-type (WT) C57BL/6 mice	1, 3, 10 μg/mL 25 mg/kg/day (20 days)	Enhanced nest-building ability (nesting score climbed from 2–3, scattered paper, to 4–5, well-organized nests within 20 days), social behavior (higher frequencies of sniffing, grooming, and following in the resident–intruder test), recognition memory (elevated recognition index in the novel-object-recognition task), and spatial learning plus reference memory (shorter escape latencies in the Morris Water Maze)	[85]

Other phytochemicals indirectly improve phagocytic efficiency by modulating inflammatory pathways and shifting microglia toward an anti-inflammatory M2 phenotype. Eugenol, the primary component extracted from *Syzygium aromaticum*, has improved cognitive function by significantly reducing Aβ deposition and enhancing microglial phagocytic activity. Eugenol’s mechanism involves suppressing inflammation and stimulating phagocytosis, which enhances lysosomal activity, subsequently mitigating neuronal damage [83]. Gastrodin, the principal active compound from *Gastrodia elata*, alleviates neuroinflammation and microglial activation by modulating the TLR4/TRAF6/NF-κB signaling pathway. Gastrodin's action includes shifting microglial polarization from a pro-inflammatory (M1) to an anti-inflammatory (M2) phenotype, directly enhancing phagocytic activity and effectively facilitating lysosomal Aβ clearance [84]. Zerumbone effectively decreases Aβ deposition by modulating MAPK signaling pathways and enhancing microglial phagocytosis, reducing inflammation, and promoting the conversion of microglia from the M1 phenotype to the M2 phenotype [85, 86]. Additionally, gossypetin improves spatial memory by augmenting microglial phagocytic activity against Aβ, promoting the M2 phenotype, which is crucial for neuroprotection [87]. Further notable phytochemicals include cycloastragenol, extracted from *Astragalus*, which targets PDE4B to promote microglial phagocytic activity, reduce senescence-associated impairments, and alleviate Aβ deposition, potentially through PDE4B/CREB/BDNF signaling [88]. In contrast, a smaller group of compounds influences Aβ clearance through mechanisms that exhibit an inhibitory effect on this process. Trichostatin A, a histone deacetylase inhibitor, upregulates the expression of albumin in the brain, which helps reduce Aβ aggregation and promotes Aβ degradation [86]. Epigallocatechin Gallate (EGCG) suppresses inflammatory signaling and reduces microglial phagocytosis [89]. Phytochemicals, particularly curcumin and cyanidin-3-O-glucoside, exhibit substantial potential in reducing Aβ burden and ameliorating clinical symptoms of AD through mechanisms involving enhanced phagocytosis.

#### Phytochemicals enhancing lysosome-mediated autophagy in AD

**Fig. 3:**
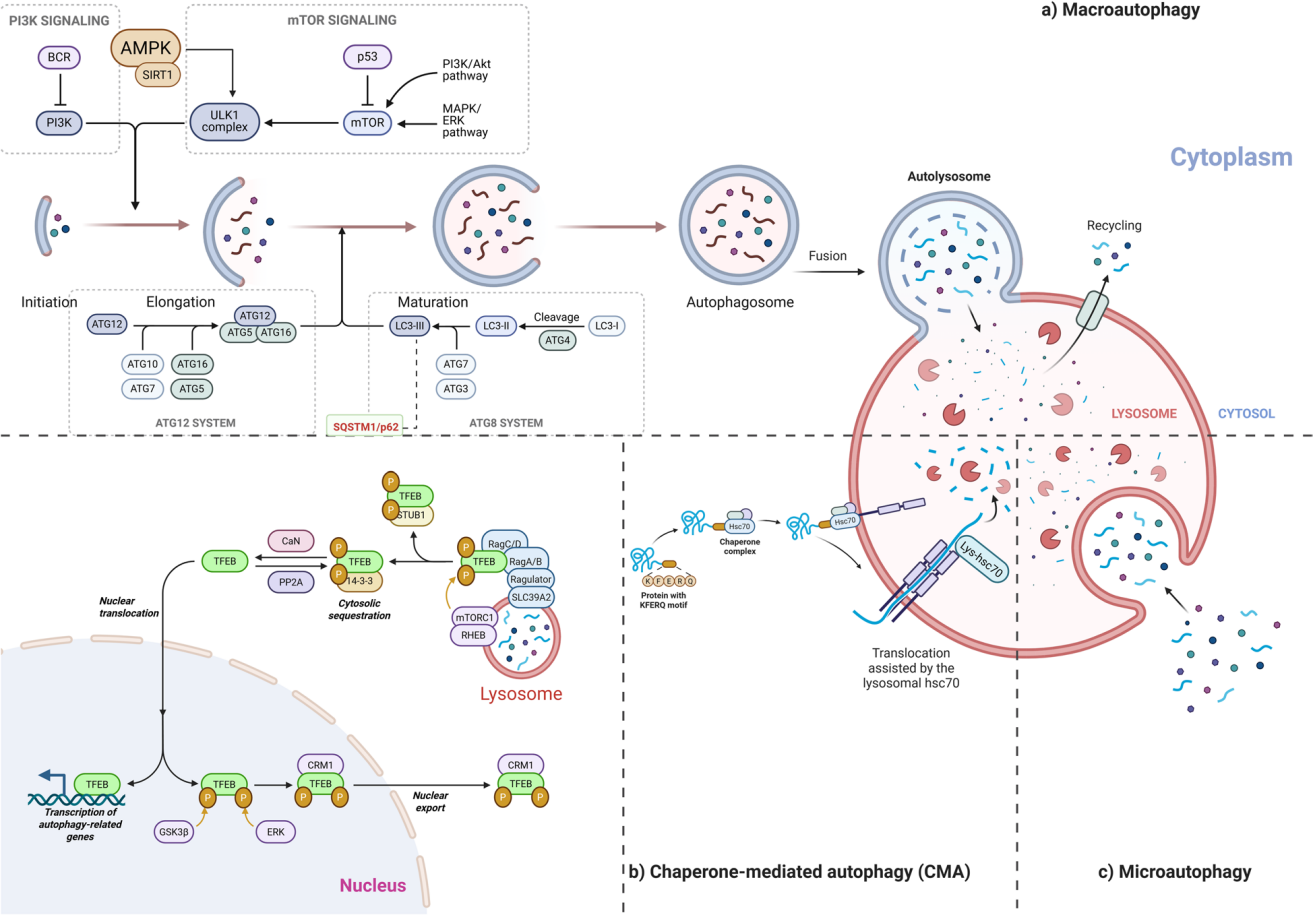
Overview of autophagy pathways

Autophagy is a lysosome-dependent cellular degradation process crucial for maintaining proteostasis and removing toxic protein aggregates in neurodegenerative diseases. The three main types of autophagy are macroautophagy (commonly referred to as autophagy), chaperone-mediated autophagy (CMA), and microautophagy – Fig. 3. Macroautophagy plays a crucial role in removing damaged proteins and organelles; it is also essential in degrading APP and its metabolic products [10, 90, 91], while CMA and microautophagy serve complementary roles in processing specific proteins or other cellular constituents [40, 92].

Atg5-dependent autophagy enhances the degradation of the APP itself [93], inhibition of Atg7 weakens the autophagic flux, leading to reduced extracellular Aβ plaque formation and increased Aβ accumulation within neurons, indicating Aβ secretion is impaired in autophagy due to dysfunctional regulation [94]. Inhibition of mTOR (mechanistic target of Rapamycin) signaling using certain identified compounds can significantly reduce tau phosphorylation and insoluble tau formation [91]. Enhancing Transcription Factor EB (TFEB) encourages autophagy and lysosomal physiology and helps accelerate APP degradation [10, 95]. Aβ accumulates in autophagosomes and can be transferred to autolysosomes for breakdown through the cathepsin D. The optineurin and proteasomal system proteins break down extracellular Aβ fibrils through the phagocytic microglia; the proteasomal system can only degrade monomeric and low-molecular-weight Aβ42 oligomers. However, autophagy can degrade in regulating the aggregation, phosphorylation, and degradation of microtubule-associated protein tau [10].

The errors in the formation of autophagosomes have been observed in AD patients, potentially due to reduced Beclin-1, which regulates the synthesis of autophagosomes [90]. Autophagy is a lysosome-dependent cellular degradation process crucial for maintaining proteostasis and removing toxic protein aggregates in neurodegenerative diseases. The three main types of autophagy are macroautophagy (commonly referred to as autophagy), chaperone-mediated autophagy (CMA), and microautophagy—Fig. 3. Macroautophagy plays a crucial role in removing damaged proteins and organelles; it is also essential in degrading APP and its metabolic products [10, 90, 91], while CMA and microautophagy serve complementary roles in processing specific proteins or other cellular constituents [40, 92].

During autophagy, LC3 undergoes lipidation and becomes attached to the inner and outer membranes of the double-membrane autophagophore before it closes. This modification not only facilitates the transport of autophagosomes and their fusion with lysosomes but also plays a key role in selecting the specific cargo for degradation, ensuring efficient clearance of damaged or unnecessary cellular components [96]. PSEN1 preserves lysosomal acidification by directing the v-ATPase to lysosomes, while PSEN2 regulates the Rab7-Ras-related protein-7 to autophagosomes by regulating Ca2 + balance in the endoplasmic reticulum. Therefore, alterations in PSEN1 and PSEN2 impair lysosomal degradation and obstruct the merging between autophagosomes and lysosomes [90, 97]. Autophagy also helps regulate inflammation by degrading the NLRP3 inflammasome and controlling factors RIPK1 and TFEB [90].

Phytochemicals targeting lysosome-mediated autophagy are demonstrated in Table 2. Compounds stimulating the AMP-activated protein kinase (AMPK) signaling axis, a central energy-sensing pathway that governs autophagy initiation [98, 99]. By activating AMPK, they inhibit the mechanistic target of rapamycin (mTOR), release the autophagy-initiating kinase ULK1, and in some cases stimulate TFEB to promote lysosomal biogenesis in enhanced autophagosome formation, improved clearance of protein aggregates, and autophagic flux restoration [99, 100]. Naringenin and kaempferol activate AMPK-mediated autophagy signaling to reduce Aβ accumulation and associated neurotoxicity, protecting neuronal cells from oxidative stress-induced apoptosis [101, 102]. Genipin, an iridoid glycoside derived from *Genipa americana L.*, exerts a potent modulatory effect on autophagy by activating the SIRT1/LKB1/AMPK signaling cascade. By stimulating this energy-sensing pathway, genipin suppresses the mTOR/p70S6K axis, thereby enhancing autophagic flux and facilitating the clearance of pathological protein aggregates [103–105]. Crocetin activates STK11/LKB1, phosphorylates AMPK at Thr172, ULK1 at Ser317, and represses mTOR. The cascade raises LC3B-II, Atg5/7/12, and BECN1, driving autophagic flux and confirming the central role of the STK11–AMPK axis. In N9 microglial cells, 25 µM crocetin lowers Aβ42; the effect vanishes when you block autophagy or knock down STK11. In mice, oral dosing at 5–20 mg/kg/day for 30 days reactivates STK11–AMPK signalling, increases hippocampal LC3B-II, cuts plaques and soluble Aβ, reduces GFAP and IBA-1, raises autophagy proteins, and improves Morris water-maze performance, an autophagy activator acting through STK11-AMPK-ULK1/mTOR to remove Aβ, suppress neuroinflammation, and improve cognition [106].

**Table 2:** Pharmacologically active compounds targeting autophagy

Group	Compounds	Structure	Species	Activities	Mechanism	Models	Dose	Behavioral experiment results	Ref
Polyphenol	2,3,5,4′-tetrahydroxystilbene-2-O-glycoside		*Polygonum multiflorum* – He Shou Wu	Dampens neuro-inflammation, restores autophagy/mitophagy flux, and provides neuro-protection	Bound the AMPK-α pocket (docking) and triggered AMPK/ ULK1 → PINK1/Parkin signalling, lowered iNOS and COX-2 levels in microglia	BV2, N2a, and SH-SY5Y neonatal rat cortex & hippocampus	10 µM, 1 µM, 100 nM, 10 nM (12–24 h)		[21]
Flavonoid	Apigenin		*Morifolium chrysanthemum* – Ju Hua	Antioxidant, anti-amyloid (Aβ-42) aggregation, induces autophagy-mediated degradation of β-catenin	Restores glutathione while lowering GST, SOD, catalase, TBARS, and protein-carbonyls, inhibiting the Akt/mTOR signaling pathway, reduces the levels of β-catenin in both the cytoplasm and nucleus, and induces the formation of autophagosomes	Transgenic *Drosophila melanogaster* P19 cells HCT-116, SW480, and WiDr cells	25, 50, 75, 100 µM 10 μM to 50 μM (16–22 h)	Improved locomotor function (increased percentage of flies crossing 8 cm within 10 s)	[168, 169]
Alkaloid	Berberine		*Coptis chinensis* – Huang Lian	Reduces tau hyperphosphorylation, enhances autophagic, protective effects on oxidative stress-induced apoptosis	Modulating the Akt/GSK3β and Protein Phosphatase 2A (PP2A) pathways, through the class III PI3K/Beclin-1 pathway, modulates endoplasmic reticulum stress by inhibiting (GRP78, caspase12, CHOP) and autophagy pathways by downregulating (LC3, Beclin-1, and p62)	3 × Tg-AD mice Human NP cells SD rat	100 mg/kg/day (4 months) 8 μM (24 h) 150 mg/kg/day (8 weeks)	Improved learning ability (reduction in the time to find the hidden platform) Improved memory retention in both short-term (24 h later) and long-term (72 h later) memory tests (mice spent more time in the target quadrant) Reduced tau accumulation in the hippocampus, specifically in the dentate gyrus and CA1 region	[170, 171]
Triterpenoid	Celastrol		*Tripterygium wilfordii* Hook F—Lei Gong Teng	Activates TFEB-mediated autophagy and lysosomal biogenesis, decreases insoluble, phosphorylated Tau aggregates	Inhibits mTORC1, causing de-phosphorylation of TFEB, increases LC3-II, LAMP1, cathepsins; more Lysotracker-positive vesicles	HeLa-CF7 (3 × Flag-TFEB), HEK293, N2a C57BL/6 J mice; P301S Tau transgenic mice; 3xTg-AD mice	0.25–1 µM (6–9 h) 1 or 2 mg/kg/day (2.5—9 months)	Enhanced associative memory, exploration, and spatial learning and reference memory (contextual-fear freezing percentages increased; open-field centre-zone time decreased with improved locomotor/exploratory activity; Morris Water Maze training escape latencies on days 1–6 shortened and probe-trial time in the target quadrant prolonged)	[172, 173]
Carotenoid	Crocetin		*Crocus sativus* L. – Xi Hong Hua	Enhances the clearance of Aβ by autophagy	Activating the AMPK pathway activates the STK11 kinase, upregulates LC3B-II, Atg7, and Atg12, while inhibiting mTOR, suppresses TNF-α, IL-1β, IL-6, and IL-8, and enhances IL-10 levels Inhibits the activation of NF-κB and reduces the expression of p53 in the hippocampus	N9 microglial cells and primary neuronal cells HeLa cells transfected with the Swedish mutant APP751 Wild-type C57BL/6 and 5XFAD APPsw transgenic mice	3.12 to 50 µM (12 h) 10, 20, and 40 μM (8 h) 10 mg/kg (30 days) 10 and 30 mg/kg/day (6 months)	Improved cognitive function (decreased escape latency and increased time spent in the target quadrant), reduced neuroinflammation (decreased astrocyte and microglial markers) in the brains of 5XFAD mice Improved memory (spent more time exploring a novel object)	[106, 174]
Iridoid glycoside	Genipin		*Genipa americana* L.—Zhi Zi	Lowers tau phosphorylation, enhances autophagic flux	Downregulates Tau kinases CDK5 and p-GSK3β, activates SIRT1/ LKB1/ AMPK, suppressing mTOR / p70S6K and autophagy	Human Tau-R3 peptide HEK293/Tau441 SH-SY5Y/Tau441 N2a/SweAPP hippocampal neurons from 3 × Tg-AD mice	5–40 µM (24 h)		[103–105]
Saponin	Ginsenoside Rg2		*Panax ginseng* – Ren Shen	Autophagy activation, lysosomal hydrolase (cathepsin L > B) activation, reduction of intracellular Aβ (1–42) load and secretion, antioxidant and mitochondrial protection	Increases LC3-II and Beclin-1 and reduces p62. Cathepsin L activity and cathepsin B are increased, ROS is decreased, and mitochondrial membrane potential is restored. Nrf2 translocates to the nucleus, driving higher HO-1, GST, and OGG1 expression	SH-SY5Y male ICR	50 µM (24 h) 10 &20 mg/kg/day (4 weeks)	Restored spatial learning and reference memory (shorter escape latencies during training, more platform crossings, and longer residence in the target quadrant on the probe day, indicating that swimming speed was unaltered)	[60, 67]
Flavonoid	Kaempferol		*Ocimum basilicum* – Luo Le	Promoting autophagy, neuroprotective, and anti-fibrillogenic antioxidants	Induces autophagy via AMPK/ mTOR/TFEB	Mouse neuroblastoma α-Syn-N2a cells	5 µM (48 h)		[102]
Butenolide	Ligustilide		*Angelicae sinensis* – Dang Gui	Inhibits the over-activation of endoplasmic reticulum stress pathways, reduces the expression, inhibits excessive autophagy, and promotes autophagic flux	Inhibits the over-activation of GRP78/PERK/CHOP signaling pathway, reduces the expression of GRP78, p-PERK, and CHOP, downregulates Beclin-1 and Atg5, and regulates the levels of LC3B-II/I and p62/SQSTM1	SH-SY5Y	12.5, 25, and 50 µM (12 h)		[65]
Flavonoid	Naringenin		*Citrus reticulata* Blanco – Chen Pi	Lowers insoluble plaques and soluble Aβ42, suppresses microgliosis, and astrogliosis	Blocks MAPK cascade (decreases p-p38, p-JNK, p-ERK1/2), down-regulates BACE1, limiting Aβ generation, AMPK/ ULK1 autophagy axis, dampens Aβ-triggered release of IL-1β, TNF-α, and IFN-γ, favouring a phagocytic, Aβ-clearing milieu, switches microglia M1 to M2 phenotype	BV2 microglia mouse Neuro2a neuroblastoma cells Primary mouse cortical neurons isolated at embryonic day 16–18 APPswe/PSEN1dE9 (APP/PS1) transgenic mice	50 mg/kg/day (3 months) 100 µM 50 µM (24 h)	Enhanced recognition and spatial memory (higher recognition index, decreased escape latency, increased time spent in the target quadrant and platform crossings, with no change in overall swim speed)	[101, 175, 176]
Nucleotide	Nicotinamide mononucleotide (NMN)		*Cinnamomum verum* J. Presl – Gui Rou	Promoting autophagy and reducing the accumulation of phosphorylated tau reduces oxidative stress and improves mitochondrial autophagy	Upregulation of Beclin-1 and LC3; Activating the Nrf2/Keap1/NQO1 signaling pathway; Decreases oxidative stress markers, MDA, and increases NQO1, enhancing the activity of NAD + -dependent enzymes like SIRT1	PC12 Cell ICR	100 μM, 200 μM, 400 μM, and 800 μM (24 h) 200, and 400 mg/kg (14 days)	Improved spatial memory (increase in spontaneous alternation) Increased object recognition (a higher discrimination index)	[177, 178]
Saponin	Pseudoginsenoside‐F11		*Panax quinquefolium* – Xi Yang Shen	Neuroprotection mitigates oxidative stress and neuroinflammation	Activates calcineurin, dephosphorylates TFEB and drives its nuclear translocation; up-regulates autophagy- and lysosome-related genes (Lamp1, Ctsd, Sqstm1, Map1lc3b), restores autophagy-lysosomal pathway homeostasis, activates the Nrf2/ARE pathway, increasing GST, SOD, and GSH while lowering H₂O₂ and MDA, reduces AGEs/RAGE signalling and NLRP3-inflammasome activation (decreased IL-1β, caspase-1, NLRP3)	Primary cortical neuron SAMP8 mice C57BL/6 mice	10 – 100 µM 6 and 12 mg/kg (IV – 1 dose) 2–16 mg/ kg/day (9 weeks)	Enhanced exploration, recognition, and spatial learning and reference memory (training escape latencies shortened; probe-trial time, distance, and crossings in the target quadrant increased; novel-object discrimination indices fully restored at 2 h and 24 h delays; step-through latency prolonged in passive-avoidance; locomotor distance and vertical rearings recovered in the open field)	[179–181]
Flavonoid	Quercetin		*Raphanus sativus* L. – Lai Fu	Lowers paired-helical-filament Tau, Aβ1-40/42 loads, and improves cognitive and emotional performance	Suppresses hyperphosphorylated Tau and amyloid pathology AMPK-dependent pathway	*Caenorhabditis elegans* BR5270 tauopathy strain 3 × Tg-AD mice	150 µM 25 mg/kg (3 months)	Extends lifespan by ≈ 8.6% and maximum survival by ≈ 2 days Better spatial learning & memory (decreases escape latency, increases target-quadrant time)	[182]
Diterpenoid	Tanshinone IIA		*Salvia miltiorrhiza –* Dan Shen	Alleviating oxidative stress, cognitive enhancement, and Aβ clearance promotion promotes autophagy	Downregulates MDA, upregulates SOD and GSH-Px, Activates DAF-16/FOXO, upregulates SOD-3, GST-4, UNC-51, increases unc-51, bec-1, atg7, lgg-1 and atg18 level; benefits abolished by 3-MA, independent of SKN-1 and HSF-1 pathways, activation of SIRT1, up-regulates LRP1 and down-regulates RAGE, decreases COX-2, iNOS, NF-κB expression; restores IκBα	bEnd.3 APP/PS1 double-transgenic mice CD-1 mice *Caenorhabditis elegans* CL2006 strain	20 µM (24 h) 10 and 20 mg/kg (8 weeks) 1, 3, 10 mg/kg (21 days) 5 μg/mL or 50 μg/mL	Improved memory and spatial learning (decreased escape latency, increased platform crossings, and greater time spent in the target quadrant) Reduced anxiety-like behavior (more entries and longer distance traveled in the central zone) Enhanced recognition memory (higher discrimination index for the novel object) Mean lifespan increased in a dose-dependent manner	[183–186]
Alkaloid	β-asarone		*Acorus tatarinowii Schott* – Shi Chang Pu	Induces autophagy, enhances learning and memory, and inhibits the accumulation of Aβ plaques	Increases LC3 I/II and BECN, increasing the expression of PINK1 and Parkin, suppresses APP, PS1, Aβ, and BACE1, while promoting synaptophysin (SYN1)	PC12 cells	12, 24, 36, 72, 144 µM (12 h)		[112, 113]

The compounds target autophagy at the lysosomal stage by activating TFEB, the master lysosomal and autophagy gene expression regulator. Promoting TFEB nuclear translocation boosts the cell’s degradative capacity, increases lysosomal enzyme production, and enhances the breakdown of pathogenic proteins and damaged organelles [107]. Pseudoginsenoside-F11 activates autophagy in four steps: it first stimulates the phosphatase calcineurin; calcineurin quickly de-phosphorylates TFEB so TFEB enters the nucleus; nuclear TFEB up-regulates the autophagy-lysosome genes Lamp1, Ctsd, Map1lc3b, and Sqstm1; the resulting lysosomal boost then clears LC3-II and SQSTM1, reopening autophagic flux [108, 109].

A subset of compounds that specialize in promoting mitophagy, the selective form of autophagy responsible for removing damaged mitochondria, involves activation of the PINK1/Parkin signaling pathway, which tags defective mitochondria for degradation, thereby maintaining mitochondrial quality control and reducing oxidative stress [110]. β-asarone, an alkaloid compound extracted from *Acorus tatarinowii Schott*, promotes neuronal health by enhancing autophagy and mitophagy. It increases the expression of key autophagy-related proteins LC3-I/II and Beclin-1 while upregulating PINK1 and Parkin, activating the mitochondrial quality control pathway. β-asarone facilitates the clearance of Aβ plaques, suppresses amyloidogenic enzymes—APP, PS1, and BACE1, and simultaneously supports synaptic function by increasing synaptophysin (SYN1) expression [111–113].

The agents directly influence the core autophagy machinery by modulating Beclin-1 and LC3, two essential proteins for autophagosome nucleation and elongation. Enhancing LC3-II formation and Beclin-1 expression facilitates the capture and degradation of toxic cellular debris, contributing to neuroprotection and protein homeostasis [114, 115]. Ginsenosides Rg1 and Rg2 increase LC3-II and Beclin-1, and decrease p62, autophagy activation is clear after 24 h at 50 µM: gets faster lysosomal turnover—cathepsin L jumps 40% and cathepsin B 22% with Rg2—so Rg2 triggers stronger autophagic clearance than Rg1, especially in the APP‑mutant cell [67].

The compound modulates autophagy indirectly by alleviating or balancing ER stress, a cellular condition that can either stimulate or inhibit autophagy depending on severity by suppressing GRP78, PERK, and CHOP, preventing autophagy dysregulation, and promoting a controlled, protective autophagic response [116, 117]. Ligustilide, a butenolide derivative from *Angelicae sinensis*, modulates autophagy primarily through its regulation of endoplasmic reticulum (ER) stress pathways by inhibiting the over-activation of GRP78/PERK/CHOP signaling. Ligustilide reduces excessive ER stress–induced autophagy while promoting balanced autophagic flux. It downregulates Beclin-1 and Atg5, adjusts LC3B-II/I ratios, and decreases p62/SQSTM1 accumulation, thereby restoring cellular homeostasis [117].

#### Tau pathology and phytochemical regulation of lysosomal function in AD

Lysosome-mediated autophagy plays a crucial role in tau clearance, in parallel with the ubiquitin–proteasome system. Impaired autophagy contributes to tau accumulation and downstream neurotoxicity. Dysfunction of the TFEB reduces lysosomal activity and promotes intraneuronal tau aggregation, accompanied by a marked decrease in the interstitial fluid [40, 118]. Primary and iPSC-derived neurons from tauopathy patients demonstrate that TFEB-secreting tau forms truncated at the microtubule-binding region through the lysosomal calcium channel TRPML1; when TFEB is absent, TRPML1 activity also declines [119].

The lysosomal system becomes disrupted when cathepsin D is removed, activating caspase enzymes to cleave tau at the C-terminus, producing a highly toxic truncated tau form, characterized by low solubility and a tendency to aggregate into harmful protein clusters, disrupting neuronal function, causing lysosomes enlargement and the loss of its natural acidity; tau fibrils inflict minimal nanoscale damage to the lysosomal membrane and weaken it, causing tau fibrils to escape. Then, the Endosomal sorting complex required for transport proteins is recruited to the lysosomal membrane to repair and protect it [120]. When tau fibrils escape, they can trigger tau accumulation at the lysosome surface in the cytoplasm, and the loss of NHE6 protein disrupts the endolysosomal system [121]. When Glucocerebrosidase function is impaired, lysosomes cannot efficiently degrade excess substances, causing tau accumulation and the accumulation of the lipid lactosylceramide [122].

ROS chemically modifies the substances, causing them to accumulate into fragments or aggregates that are more toxic than their original forms [12]. Autophagic mutations in the PSEN1 or PSEN2 genes impair lysosomal degradation, a common cause of early familial AD. Mutations result in lysosomal storage disorders, which share phenotypic features with AD, including neural accumulation of lysosomal vesicles. In AD, the action of cathepsins B and D is often reduced, impairing the breakdown of abnormal proteins and resulting in the intracellular build-up of Aβ [123]. Initiation of the JNK (c-Jun N-terminal kinase) and MAPK pathways contributes to cell death and inflammation [29]. When Aβ damages the lysosomal membrane and LAMP2A and Rab7 do not function properly, the merging between lysosomes and autophagosomes is hindered [124, 125].

Compounds that target the recovery of lysosomal function can effectively ameliorate early pathological manifestations of AD, as can be found in Table 3. Studies in animal models have shown that impaired lysosomal acidification precedes pathological manifestations and is considered an early marker of neurodegeneration [126]. Compounds stimulate lysosomal biogenesis and enhance the expression of LAMP1 and LAMP2, as well as hydrolytic enzymes like cathepsins D and L. They exert their effects by activating the nuclear receptor PPARα, which promotes the nuclear translocation of TFEB, a master regulator of lysosomal gene expression, strengthens the autophagic flux, and supports efficient degradation of cellular waste materials [127, 128]. 3,14,19-triacetyl andrographolide (ADA), a specialized derivative of *Andrographis paniculata* (Burm. F) Nees, notably enhances lysosomal function by increasing colocalization between the autophagy marker LC3 and lysosome-associated membrane protein 1 (LAMP1), indicating enhanced completion of lysosomal digestion and removal of damaged cellular structures, reducing toxic protein loads within neuronal cells, ADA also supports synaptic protection and improves cognitive function [129]. Cinnamic acid prominently activates TFEB, enhancing lysosomal efficiency and reducing toxic protein accumulation, induces lysosomal biogenesis, enhances lysosomal functions, leading to increased lysosomal markers, by activating the PPARα, increases LAMP2, and improves autophagy (via LC3B), binds to PPARα, and enhances the transcription of TFEB [130]. Oleoylethanolamide, an endogenous lipid amide, strongly activates the TFEB pathway, and oleoylethanolamide significantly enhances lysosomal degradation capacity, efficiently managing abnormal proteins and cellular structures, while concurrently reducing neuroinflammation commonly observed in AD [62]. In addition to the principal compounds, other substances have positively affected lysosomal function, reducing lysosomal membrane permeability and activating AMPK and SIRT1, which facilitate the lysosomal degradation of abnormal proteins – resveratrol, urolithin A [61, 131–135]. Compounds represent promising therapeutic strategies through their potent enhancement of lysosomal function, including promoting lysosomal biogenesis, boosting enzymatic activity, improving lysosomal-autophagosomal fusion, maintaining lysosomal stability, and increasing the degradation of pathogenic proteins, multifaceted actions effectively address pathological features and cognitive symptoms characteristic of AD, paving the way for developing safe and effective treatments from natural sources.

**Table 3:** Pharmacologically active compounds modulating lysosomal function

Group	Compounds	Structure	Species	Activities	Mechanism	Models	Dose	Behavioral experiment results	Refs
Diterpene lactone	3,14,19-Triacetyl Andrographolide		*Andrographis paniculata* – Chuan Xin Lian	Activates autophagy in the brain, restores lysosomal function	Activates autophagy through the Akt/mTOR pathway, decreases p-Akt and p-mTOR levels in the hippocampus and cortex, restores lysosomal function by the degradation of autophagosomes (reducing cathepsin B expression and increasing the co-localization of LC3 and LAMP1	3 × Tg-AD Mice	5 mg/kg (21 days)	Improved spatial learning and memory (reduced escape latency and increased the number of platform crossings Enhanced recognition memory in the novel object recognition test	[129]
Carboxylic acid	Cinnamic acid		*Cinnamomum cassia* Presl – Rou Gui	Induces lysosomal biogenesis, enhances lysosomal functions, leading to increased lysosomal markers	Activating the PPARα increases LAMP2 and improves autophagy (via LC3B), binds to PPARα, and enhances the transcription of TFEB	Mouse primary astrocytes and neurons 5 × FAD mouse	50, 100, 200 µM (24 h) 100 mg/kg/day (30 days)	Enhanced better spatial memory and improved memory accuracy (reduced latency to reach the goal box and fewer errors The observed memory improvement was not due to changes in physical activity (the open field test showed no significant changes in general locomotor activity (velocity, total distance, movement duration)	[130]
Alkaloid	Corynoxine and Corynoxine isomers—Fe65-EXO-Cory-B		*Uncaria rhynchophylla* (Miq.) Jack's – Gou Teng	Promoting autophagy and lysosome biogenesis to reduce Aβ accumulation	Increases levels of LC3-II and the degradation of APP and APP-C-terminal fragments, increases mature cathepsin D and LAMP1 levels. Induced the nuclear translocation of the TFEB	N2aSwedAPP Tg2567 mice 5xFAD mice	20 mg/kg/d (2 months)	Improved motor coordination and balance (increased time spent on the rotating rod) Enhanced locomotor activity and exploration (increased time spent in the center of the open field) Improved spatial learning and memory (reduction in the time to find the hidden platform) Enhanced Associative Learning and Memory (increased freezing behavior in response to the cue tone)	[187]
	Oleoylethanolamide			Increases expression of genes in lipid homeostasis, regulators of lysosomal function, microglial phagocytosis, and suppression of neuroinflammation	Activation of PPARα via its stable analog KDS-5104 increased the expression of genes involved in lipid homeostasis, including CYP4A, enhanced TFEB, enhances lysosomal biogenesis through a mTORC1-independent pathway, increases the microglial uptake of Aβ plaques dependent on PPARα and is mediated by the receptor CD36, reduced lipid droplet accumulation in microglia, and reduced the inflammatory response induced by LPS	Primary microglial cultures, BV2 cells, and HeLa cells 5xFAD mice	10 mg/kg (2 months)	Cognitive Improvement (increased exploration of novel objects and improved associative learning)	[62]
Polyphenol	Resveratrol		*Cuspidatum polygon* – Hu Zhang	Lowers brain Aβ and hyper-/acetyl-phosphorylated tau; boosts cellular protein-clearance capacity	Lowering BACE1 and raising neprilysin, higher 20S proteasome subunits and trypsin-like activity, normalised ubiquitinated proteins and Hsp70, activate the AMPK–SIRT1 signalling cascade, by increased phosphorylated AMPK, reduced acetylated p53, and higher phosphorylated CREB and PGC-1α, promote tau clearance through SIRT1-dependent, curbing NF-κB transcriptional activity and down-regulating BACE1 and Aβ generation, decreases LC3 and Beclin-1 level, reduces cathepsin D	SH-SY5Y Male 3xTg-AD mice	25 µM (48 h) 50 mg/kg/day (12 months) 100 mg/kg/day (10 months)	Enhanced exploration, recognition, and spatial learning (recovered locomotor and centre-zone exploration with quicker head-dip initiation, higher novel-object preference with a fully restored discrimination index, and probe-trial time and distance in the target quadrant)	[131–133]
Disaccharide	Trehalose		*Saccharomyces cerevisiae* – Jiao Mu	Stimulates autophagy, promotes the clearance of neurotoxic misfolded proteins, and is a potent modulator of progranulin expression, which is crucial for lysosomal function	Promoting the nuclear translocation of TFEB; also triggers lysosomal enlargement and transient membrane permeabilization, releasing calcium ions and activating PPP3CB/calcineurin, promotes the clearance of amyloid plaques and tau aggregates, increasing lysosomal pH, and inhibiting mTORC1 signaling	NSC34 motoneuronal cell primary murine macrophages, HEK 293 T and NIH 3T3	100 mM (48 h) 0.1 mM to 10 mM (24 h)		[148, 149, 188]
	Urolithin A		Urolithin A is converted from ellagitannin.​	Reduction of Aβ and tau pathologies, promotes mitophagy, and restoration of lysosomal function, anti-inflammatory, improves lysosomal function, induces autophagy	Regulating cathepsin Z levels, reducing IL-1β levels in the hippocampus and cortex, improves lysosomal function, reducing lysosomal size and permeability	APP/PS1, 3xTg-AD, 3xTg-AD/Polβ + / −	200 mg/kg/day (5 months) 25 mg/kg (10 months)	Improved learning ability (reduction in time to find the hidden platform) Improvement in memory ability (increase in spontaneous alternation) Enhanced ability to recognize novel objects (increase in recognition index) Improved olfactory function (decrease in latency to find the buried food) Enhanced long-term potentiation in the hippocampus	[61, 134, 135]

#### Phytochemicals targeting lysosomal regulation of neuroinflammation in AD

In AD, lysosomal dysfunction in microglia increases cytokine release, attracts immune cells, and activates harmful interactions with astrocytes, exacerbating inflammation. Additionally, contributing factors to the disorder are presenilin mutations, cytokine stimulation, lipid disorders, ATP signaling disruption, impaired lysosomal acidification, enhanced inflammatory responses, and cellular imbalance. Treatments to restore lysosomal acidification include small molecules and nanoparticles targeting v-ATPase, TFEB, and mTOR inhibition [8]. When lysosomal function is impaired, Aβ accumulates, leading to microglial activation, particularly in the hippocampus region, increasing CD68 and CD16/32 (markers of pro-inflammatory M1 microglia) [136]. Dysfunction or disruption of autophagy can lead to mitochondrial dysfunction and elevated production of mitochondrial ROS [137].

Phytochemicals have demonstrated potential for treating AD by targeting oxidative stress reduction and neuroinflammation suppression (Table 4). Oxidative stress leads to structural and functional damage to neurons through the accumulation of ROS and disruption of endogenous antioxidant defenses [138]. Antioxidant compounds act by neutralizing ROS, enhancing the activity of SOD, GSH-Px, and GPx4, and activating protective signaling pathways including Nrf2/HO-1, Akt/mTOR, and SIRT3, prevent neuronal degeneration, support synaptic function, and improve learning and memory performance. Depolymerized proanthocyanidins derived from peanut skins significantly inhibit Aβ42 aggregation, reduce Aβ-induced cytotoxicity, and alleviate cognitive dysfunction, leveraging their antioxidant properties for therapeutic neuroprotection [139]. Baicalein acts as an inhibitor of BACE1 and acetylcholinesterase, crucial enzymes for Aβ aggregation, and demonstrates strong antioxidant properties with effective blood–brain barrier permeability, underscoring its neuroprotective potential [63]. Crocin inhibits Aβ-induced neuronal apoptosis by suppressing intrinsic apoptotic pathways and caspase-3 activation. Its potent antioxidant effects reduce oxidative stress, protecting neurons and enhancing mental functions [140]. Salidroside improves mitochondrial function and reduces oxidative stress through the Nrf2/SIRT3 pathway, inhibiting Nrf2 degradation, facilitating SIRT3 transcription, and protecting mitochondria, effectively countering neurite dystrophy and cognitive dysfunction [141].

**Table 4:** Antioxidant and anti-inflammatory pharmacologically active compounds

Group	Compounds	Structure	Species	Activities	Mechanism	Models	Dose	Behavioral experiment results	Refs
Coenzyme	Alpha-lipoic acid		*Brassica oleracea –* Gan Lan	Inhibit Tau hyperphosphorylation and ameliorate neuroinflammation, oxidative stress, and ferroptosis	Reducing ROS and increasing Gpx4 and SOD1. Lowered TNF-α, IL-1β, and GFAP. Via the calpain1, GSK3β (Glycogen Synthase Kinase-3β), CDK5, and MAPK pathways. Inhibiting excessive calpain1 activation	P301S Tau transgenic mice	3 mg/kg and 10 mg/kg (10 weeks)	Improved memory and spatial learning (reduced latency to find the hidden platform and increased time spent in the target quadrant) Enhanced memory formation (better exploration of the novel object) Improved exploration, suggesting potential anti-anxiety effects	[189–192]
Flavonoid	Baicalein		*Scutellaria baicalensis* (Radix) – Huang Qin	Reduces intracellular Aβ42, protected from Aβ42-induced oxidative damage, exhibits anti-inflammatory properties	Lowers Aβ42-induced reactive-oxygen species, consistent with known antioxidant/Nrf2-activating capacity, inhibits the activation of poly (ADP-ribose) polymerase-1, inhibits BACE1 in a non-competitive manner	Saccharomyces cerevisiae BY4743 Δahp1 J20 transgenic mice	1–50 µM 80 mg/kg/day (6 months)	Improved locomotor function (reducing hyperactivity) Enhanced spatial memory (an increased frequency of quadrant crossings) Restored cerebral blood flow to normal levels in the brain	[63, 193–195]
Phenolic diterpene	Carnosic acid		*Rosmarinus officinalis* – Mi Die Xiang	Induction of phosphorylated tau accumulation	Reactivates IRS-1 → Akt, suppresses GSK3β activation, cuts ApoE & p-tau, and reinstates Akt/CREB/ERK-driven synaptic plasticity	APPPS1 transgenic mice	1 μM 5—20 mg/kg (10 weeks)	Enhanced exploration, recognition, and spatial working memory (restored locomotor activity and centre exploration, elevated novel-object preference with a higher discrimination index, and increased spontaneous-alternation percentage)	[145, 196]
Carotenoid	Crocin		*Crocus sativus* – Xi Hong Hua	Reduces oxidative stress	Regulating ROS, SOD, and GSH-Px levels increases p-Akt,p-mTOR	HT22 cells BALB/c Wistar	0.5 µM to 2 µM (3 h) 5 mg/kg to 20 mg/kg (4 weeks) Intra-hippocampal: 150, 300, and 600 nmol/side Intra-peritoneal: 30 mg/kg (20 days)	Improved memory (reduced escape time to the hidden platform) Improved coordination and exploration (displayed more purposeful movement and reduced chaotic wandering)	[197, 198]
Flavonoid	Hesperetin		*Citrus reticulata* Blanco – Chen Pi	Antioxidants, anti-inflammatory	Activates the Nrf2, HO-1 axis, Suppresses the TLR4/NF-κB	HT22 neurons & BV-2 microglia C57BL/6N mice	50 µM (24 h) 50 mg/kg	Improved spatial learning and working memory (escape latency shortened; platform crossings and time in the target quadrant increased; spontaneous-alternation percentage elevated; swim speed unchanged)	[155, 199]
Alkaloid	Huperzine A		*Huperzia serrata* – Shi Shan	Improves cognitive function	Downregulating proteins related to iron intake (TfR1) and increasing the expression of iron-export proteins (FPN1) reduces the generation of ROS by inhibiting NADPH oxidase (NOX 2 and NOX 4)	C57BL/6	0.1 mg/kg (21 days)	Improved memory and learning (decreased escape latency and distance traveled) reduced anxiety-like behavior (increased time and distance in the center area)	[200]
Flavonoid	Luteolin		*Scutellaria baicalensis* – Huang Qin	Neuro-protective, anti-neuro-inflammatory, lowers cortical Aβ plaque	Activation of NF-κB and MAPK; decreases TNF-α, IL-1β, IL-6, NO and COX-2, iNOS, activation of PPARγ	3 × Tg-AD mice Rat C6 glioma cells	20 & 40 mg/kg/day (3 weeks) 1–10 µM (24–48 h)	Enhanced spatial learning and memory (decreased escape latency, increased time spent in the target quadrant and platform crossings, with no change in overall swim speed)	[201, 202]
	Osmundacetone		*Rhizoma Osmundae* – Zi Qi	Inhibition of Aβ fibrillation, promotion of lysosomal degradation of Aβ, reduction of oxidative damage, suppression of neuroinflammation	Binds to Aβ, preventing the aggregation of Aβ monomers into toxic fibrils. After binding to Aβ, it facilitates its lysosomal degradation, increases the expression of Gpx4, and inhibits the phosphorylation of p65	APP/PS1 transgenic mice	1 mg/kg (12 weeks)	Improvements in spatial learning and memory (shorter path lengths and latencies in the hidden platform phase of the Morris water maze)	[146]
Polyphenolic	Proanthocyanidins		*Cinnamomum verum* – Rou Gui	Suppresses Aβ42 fibril formation, exerts strong antioxidant capacity and anti-inflammatory action, and lowers plaque burden in the hippocampus	Dock onto Aβ42 through multiple hydrogen bonds and π–π stacking, blocking nucleation and elongation; their radical-scavenging activity eases oxidative stress, while IL-6, IL-1β, and TNF-α are down-regulated	SH-SY5Y SD rat	50 – 200 µg/mL 100 mg/ kg/day or 400 mg/ kg/day (50 days)	Enhanced spatial learning and reference memory (escape latency shortened on day 3 (low dose) and day 5 (high dose); probe-trial crossings of the former platform increased)	[139]
Flavonoid	Salidroside		*Rhodiola rosea* – Hong Jing Tian	Protects neurites and mitochondria from Aβ, reduces Aβ plaque burden in the hippocampus	Binds directly to Nrf2, blocks its interaction with KEAP1, stabilising and driving Nrf2 into nuclei, Nrf2 transcriptionally up-regulates SIRT3, lowered ROS, preserved MMP	SH-SY5Y Primary cortical neurons from Sirt3foxp mice 5 × FAD transgenic mice 5 × FAD + hippocampal SIRT3 knock-down	50 µM (24–72 h) 0.3 mg/kg (3 months)	Enhanced spatial learning and memory (decreased escape latency across training days, increased time and distance in the target quadrant on the probe trial, and more entries and distance travelled in the novel arm of the Y-maze, with no change in overall swim speed)	[141, 203, 204]
Isothiocyanate	Sulforaphane		*Brassica oleracea var. italica)* – Xi Lan Hua	Decreases Aβ generation, antioxidant, anti-inflammatory, and enhances cell viability	Reactivation of the Nrf2 pathway	Mouse neuroblastoma N2a/APP	1.25—5 µM (24–72 h)		[205–207]
Xanthone	α-Mangostin		*Garcinia mangostana* – Shan Zhu	Antioxidative, anti-inflammatory, and neuroprotective effects, inhibiting microglial activation, reducing the production of pro-inflammatory cytokines and NO	Inhibiting the TLR4/TAK1/NF-κB signaling pathway, reducing the activation of TNF-α, IL-6, and iNOS	BV-2 cells C57BL/6 mice	100—500 nM 50 mg/kg/day (14 days)	Improvements in spatial learning and memory (shorter escape latencies and more platform crossings) Did not affect motor function but improved cognitive abilities (No significant changes in locomotor activity)	[147]

In addition to antioxidant compounds, there are also compounds that possess both anti-inflammatory and antioxidant properties. The overactivation of microglia and astrocytes often triggers chronic neuroinflammation in response to Aβ, abnormal tau, and oxidative stress. Neuro-anti-inflammatory compounds exert their effects by inhibiting pro-inflammatory signaling pathways—NF-κB, MAPK, and TLR4/TAK1, thereby reducing the production of pro-inflammatory cytokines (TNF-α, IL-1β, IL-6) and inflammatory enzymes (COX-2, iNOS). By controlling neuroinflammatory responses, these compounds help protect neurons, maintain a stable neural microenvironment, and limit the spread of damage within the brain [142–144]. Carnosic acid acts at the interface of mitochondrial dysfunction and neuroinflammation, reducing oxidative stress and inflammation via activating Nrf2 and enhancing the Nrf2/HO-1/NQO1 pathway, inhibiting NF-κB signaling and NLRP3 inflammasome activation, thus preserving mitochondrial function and promoting autophagic and mitophagic pathways to clear damaged mitochondria [145]. A recent study highlights osmundacetone, a phytochemical extracted from *Rhizoma Osmundae*, which directly binds to Aβ, inhibiting its fibrillation process, and also promotes its lysosomal degradation, alleviates oxidative damage by increasing Gpx4 expression and suppressing neuroinflammation by inhibiting NF-κB phosphorylation, enhancing lysosomal clearance of Aβ [146]. α-mangostin attenuates inflammation by inhibiting the TAK1/NF-κB pathway, similar to gastrodin, thereby supporting neuroprotective functions [147].

#### Network-based insights into phytochemical modulation of lysosomal dysfunction in AD

Lysosomal dysfunction in AD involves complex crosstalk among amyloid deposition, oxidative stress, and impaired autophagy signaling. To systematically assess the multi-target potential of phytochemicals, a compound–protein interaction network was constructed encompassing three major modules: natural bioactive compounds, inflammation, oxidase stress-related pathogenic mediators, and intracellular autophagy–lysosome regulatory proteins.

The phytochemical constituents targeting lysosomal dysfunction in AD were identified from peer-reviewed literature and natural product databases. The chemical structural information for each compound was retrieved from the PubChem database. Potential protein targets were predicted using the SwissTargetPrediction platform, and only targets with probability scores greater than zero were included. Redundant entries were standardized and removed using the UniProt database. AD–related targets were obtained through systematic searches of the GeneCards databases using the keyword “Alzheimer Disease.”

A “compound–regulatory protein” network was constructed in Cytoscape 3.9.1 to reveal interactions among natural bioactive compounds, inflammatory mediators, oxidase stress mediators, and autophagy–lysosome pathway regulators. The integrated network highlighted two major high-degree clusters: (i) key natural compounds (magnolol, trehalose, salidroside, crocetin, genipin) and (ii) neuro-inflammation/neurodegeneration-related nodes (Aβ, IL-6, IL-1β, TNF-α, ROS). After excluding free nodes, network topology parameters were analyzed in Cytoscape, and core targets were predicted based on median thresholds of betweenness centrality, closeness centrality, and degree, with ranking performed by degree values meeting the criteria were visualized to elucidate the biological processes and signaling pathways implicated in the multi-target actions of these phytochemicals against lysosomal dysfunction in AD.

**Fig. 4:**
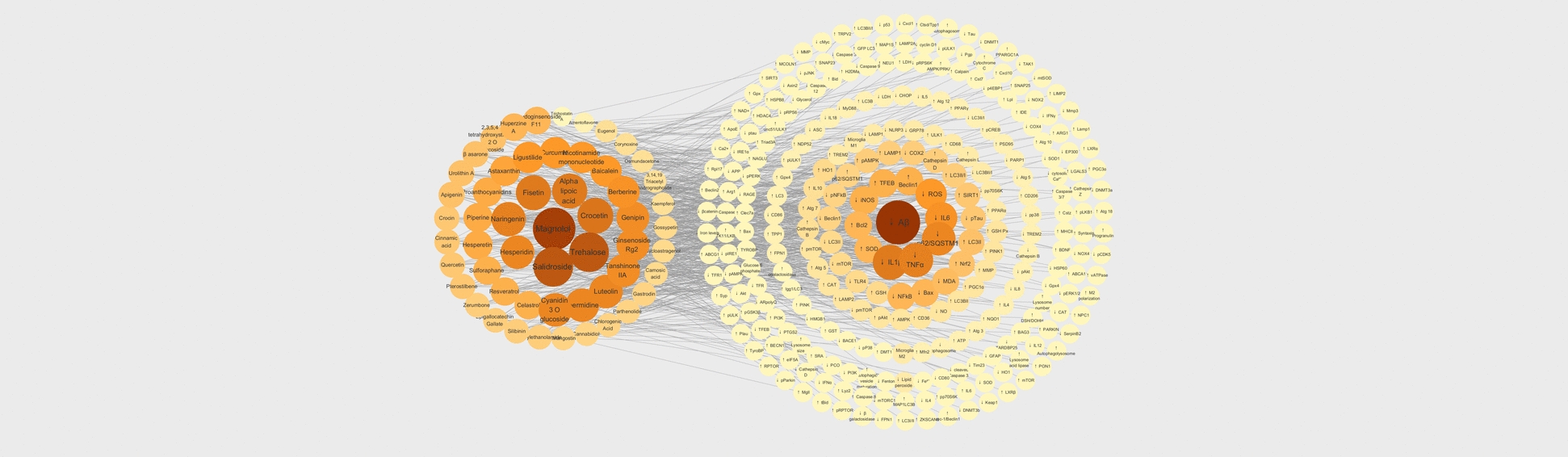
Network Map of Phytochemical–Protein Interactions Targeting Lysosomal Dysfunction and anti‑inflammatory/antioxidant pathways for Alzheimer Disease therapy. Cluster 1 (left, ~ 50 nodes; orange gradient) groups phytochemicals that exhibit lysosome‑restorative, anti‑inflammatory, and/or antioxidant activity. Cluster 2 (right, ~ 45 nodes; darker orange‑brown) contains key pathological and signalling mediators enriched in lysosomal and autophagy‑related proteins/enzymes. Notes: “↑” represents the up-regulated targets, “↓” represents the down-regulated targets

Modularity analysis was performed on the network into two clusters (Fig. 4). Cluster 1 contains 46 phytochemicals; magnolol, trehalose, and salidroside are central hubs. Cluster 2 contains key pathological and signalling mediators (Aβ, IL‑6, IL‑1β, TNF‑α, ROS) and encompasses 112 intracellular lysosome-related-pathway proteins (Beclin‑1, LC3‑II, mTOR, ULK1, p62, AMPK) and forms the principal “bridge” linking Clusters 1. The pathogenic cytokine is influenced indirectly via the lysosome-related proteins of Cluster 2, highlighting a multi‑target, multi‑layer interaction pattern that could be exploited for therapeutic intervention in neurodegenerative disorders.

Figure 4 illustrates the mechanisms underlying disease treatment through natural bioactive compounds and their impacts on crucial biological pathways involved in inflammation, oxidative stress, and autophagy. Recent studies have emphasized phytochemicals such as magnolol, salidroside, and trehalose in treating chronic inflammation and oxidative stress diseases. Phytochemicals help cells eliminate damaged proteins and minimize the accumulation of toxic elements, thereby ameliorating aging-related disorders and tissue damage.

Traditional medicinal herbs containing active compounds commonly exhibit the capability to reduce Aβ, a critical component in the progression of AD. Additionally, many natural compounds demonstrate effectiveness in modulating Beclin-1, TFEB, and p62, key regulators in autophagy, as well as reducing inflammation markers—iNOS, IL-6, IL-1β, TNF-α, and ROS. However, the capacity to regulate lysosomes through Cathepsin and LC3II is inconsistent and not universally exhibited. Only a few compounds (resveratrol and oleoylethanolamide) have shown the ability to influence lysosomal quantity, autophagosomes, or lysosomal integrity, although existing research remains limited. Compounds recently attracting less attention regarding their potential roles in treating AD through lysosomal pathways include trichosanthin A and amentoflavone, suggesting further research to elucidate their therapeutic potential.

Several phytochemicals have also demonstrated remarkable potential in regulating biological factors related to oxidative stress and inflammation, aiding in modulating inflammatory markers and reducing cellular damage through signaling pathways—NFκB, Nrf2, and AMPK. On the other hand, certain less-known compounds, including zerumbone, gastrodin, and crocin, have shown promising preliminary effects. However, scientific evidence regarding their direct influence on lysosomal mechanisms remains limited in AD treatment (e.g., LAMP2A, LIMP2, MCOLN1). Most research has focused on autophagy, overlooking substances capable of directly improving lysosomal functions and components that do not directly interact with lysosomes.

Magnolol, a natural neolignan derived from *Magnolia officinalis—*Houpo, substantially reduces Aβ accumulation and toxicity by activating the nuclear receptor PPAR-γ. Magnolol promotes microglial phagocytosis and Aβ degradation by enhancing ApoE expression via the PPAR-γ and LXR (Liver X Receptor) pathways, thus facilitating efficient lysosomal clearance [64]. Trehalose, a naturally occurring disaccharide, activates autophagy without mTOR suppression by accumulating in lysosomes, causing brief membrane permeabilisation that releases Ca^2^⁺, triggers calcineurin, dephosphorylates TFEB, and up-regulates BECN1, p62, and LC3; the leak closes within hours via lysophagy, keeping stress low. In AD mouse models, systemic or intracerebral trehalose lowers Aβ production, reduces plaque load, and restores learning and memory in APP/PS1 and APP23 strains. By speeding clearance of misfolded Aβ and tau while sparing mTOR signalling, trehalose provides a direct metabolic route to disease-modifying therapy [148]. Trehalose exerts neuroprotective effects by markedly elevating progranulin expression. It is essential for maintaining lysosomal stability and efficiency, sustaining their degradative capacity, and reducing neuronal damage caused by cellular waste [149].

In addition to the aforementioned potential compounds, there are other multi-target compounds that modulate pathways related to lysosome–autophagy, anti-inflammatory, and antioxidant activities (Table 5). Pterostilbene supports neuronal autophagy; when 3-MA blocks autophagy, pterostilbene reactivates LC3-dependent clearance, raising Aβ endocytosis from ≈ 15% to 34–75% of control. mRFP-GFP-LC3 imaging shows more red LC3 puncta, marking stronger autophagic flux. The compound does not increase surface TLR4 but drives LC3-associated endocytosis, directing Aβ to lysosomes faster and lowering amyloid burden and neuroinflammation in APP/PS1 mouse brain [150]. Pterostilbene also exhibits anti-inflammatory properties through mediating Triad3A-dependent ubiquitination and degradation of TLR4, subsequently inhibiting neuroinflammation pathways and significantly mitigating cognitive impairment [150, 151]. Chlorogenic acid, a common phenolic compound, positively modulates lysosomal activity by significantly upregulating the expression and activity of cathepsin D, similar to that of resveratrol, effectively increasing lysosomal digestion of Aβ, thereby reducing amyloid accumulation [133, 152]. Docosahexaenoic acid-acylated astaxanthin monoester significantly promotes autophagosome-lysosome fusion, thereby restoring lysosomal degradation capacity, effectively reducing the accumulation of Aβ in neuronal cells [153, 154]. Astaxanthin targets the SIRT1/PGC-1α signaling pathway, effectively mitigating neuronal senescence and apoptosis caused by oxidative stress. Astaxanthin reduces neuronal apoptosis by enhancing mitochondrial function and reducing ROS [154]. Hesperidin enhances neuronal survival and cognitive functions by inhibiting inflammation, oxidative stress, and apoptosis by reducing TLR4, HMGB1, and NF-κB signaling pathways [155]. Fisetin facilitates the degradation of phosphorylated tau through activation of autophagic pathways regulated by TFEB and Nrf2, promoting clearance of tau aggregates and enhancing autophagic lysosomal functions for neuronal health [156]. Fisetin activates autophagy by boosting LC3-II and Beclin-1 and driving the AMPK–SIRT1 pathway; at the same time, it cuts Aβ and phosphorylated tau, raises neprilysin to clear amyloid, and restores synaptic proteins (SNAP-25, PSD-95, p-CREB, and p-CaMKII), leaving you with less toxic build-up, lower inflammation, and steadier neurotransmission [157]. Cannabidiol enhances microglial phagocytosis of Aβ by activating the TRPV2 channel and promoting autophagy via the PDK1/Akt pathway, simultaneously attenuating neuroinflammation and improving mitochondrial function [158]. Lastly, parthenolide suppresses microglial-mediated neuroinflammation by modulating MAPK/TRIM31/NLRP3 pathways, significantly enhancing phagocytosis and reducing inflammatory cytokines, contributing to improved cognitive outcomes [159].

**Table 5:** Pharmacologically active compounds targeting multiple pathways

Targeting	Group	Compounds	Structure	Species	Activities	Mechanism	Models	Dose	Behavioral experiment results	Refs
Autophagy + antioxidant	Carotenoid	Astaxanthin—monoester AST docosahexaenoic acylated acid		*Haematococcus pluvialis* – Hong Qiu Cao	Reducing cognitive deficits, an antioxidant, enhances autophagic flux by restoring autophagy-lysosome fusion	Activating the SIRT1/PGC-1α signaling pathway, induces AMPK phosphorylation, activates ULK1 at Ser555 (pro-autophagic) while inhibiting mTOR at Ser757, enhances the fusion of autophagosomes with lysosomes	PC12 cells SH-SY5Y ICR APP/PS1 transgenic	10 μM (48 h) 25–50 μg/mL (24 h) 10 mg/kg (30 days) 30 mg/kg/day (3 months)	Improved the cognitive function (decreased escape latency and increased time spent in the target quadrant)	[153, 154]
Phagocytocis + Autophagy	Cannabinoid	Cannabidiol		*Cannabis sativa* – Da Ma	Enhances microglial phagocytosis and clearance of Aβ, reduces neuroinflammation, promotes autophagy, and improves mitochondrial energy metabolism	Upregulation of phagocytic receptors (Trem2, GPR34, CR3, P2Y6), activation of the PDK1/Akt pathway, induction of autophagy (Increases LC3B-II, p62, Beclin-1), enhancement of mitochondrial function and ATP production, reduction of pro-inflammatory cytokines	Primary mouse microglia and BV2 APP/PS1 transgenic	5 µM (12–24 h)		[158]
Autophagy + lysosomal function	Phenolic acid	Chlorogenic Acid		*Lonicera japonica* – Jin Yin Hua	Alleviates cognitive deficits and neuronal damage by modulating autophagy and enhancing lysosomal function	Via the mTOR/TFEB signaling pathway, suppressed autophagic processes, by reduced LC3B-II/LC3B-I and p62/SQSTM levels, decreased the expression of Beclin-1 and Atg5, increased LysoTracker Red fluorescence, and elevated levels of cathepsin D	SH-SY5Y APP/PS1 double transgenic mice	6.25–50 µM 40 mg/kg/day (6 months)	Improved spatial memory (reduced escape latency and increased time spent in the target quadrant)	[133, 152]
Autophagy + anti-inflammation	Flavonoid	Fisetin		*Gleditsia sinensis* Lam – Zao Jiao Ci	Promotes autophagy, decreases amyloid and tau, and has anti-inflammation effects, lowers phosphorylated-tau levels, and clears sarkosyl-insoluble tau aggregates	Decreases IL-6, TNF-α, NF-κB, increases Beclin-1, LC3-II, Activates AMPK/SIRT1 signalling, suppressing the TLR4 / MyD88/NF-κB, inhibits mTORC1 → nuclear translocation of TFEB, up-regulates autophagy/lysosomal genes (atg9 b, LAMP1), activates Nrf2, increases selective-autophagy receptors	Mouse cortical neuron line T4 Rat primary cortical neurons HEK-293 cells ICR mice	5 – 20 µM (24–36 h) 25–50 mg/kg/day (4 weeks)	Improved locomotor function (hyperactivity curtailed, with fewer rearings) Enhanced memory retention (increases step-down latency, decreases error shocks)	[156, 157]
Autophagy + anti-inflammation	Flavonoid	Hesperidin		*Citrus reticulata* Blanco – Chen Pi	Restores cognitive performance, anti-inflammatory antioxidant, enhances hippocampal neurogenesis, reduces Aβ burden, and rescues cognitive deficits	Downregulates HMGB1, TLR4/RAGE axis, NLRP3 inflammasome, NF-κB, and downstream IL-1β, IL-8, IL-18, MCP-1, restores Nrf2 signalling and CAT, SOD, PON-1, suppresses overactive PI3K/Akt/mTOR cascade, increases LC3-II, Beclin-1, activates the AMPK/BDNF/TrkB/CREB axis, upregulates AMPK/CREB signalling	Primary neural stem cells from embryonic mouse cortex & hippocampus Wistar rats 5xFAD transgenic mice	10–200 µM 80 mg/kg/day (7 days) 100 mg/kg (2 months)	Enhanced recognition and spatial memory (increased sniffing time and discrimination index, decreased escape latency, increased time spent in the target quadrant and distance travelled in the target quadrant, while overall locomotor activity and rearings were unchanged)	[208, 209]
Autophagy + Antioxxidant	Polyphenol (Lignan)	Magnolol		*Magnolia officinalis* – Hou Po	Lowers amyloid burden, promotes autophagy flux, inhibits apoptosis, and ameliorates cognitive decline	Activates PPAR-γ, activates the AMPK/mTOR/ULK1, leading to LC3-II & Beclin-1 accumulation and p62 degradation; simultaneously down-regulates Bax & cleaved-caspase-9 and up-regulates Bcl-2, down-regulating iNOS, IL-1β and TNF-α and activates the Nrf2-ARE pathway	N2a BV2 APP/PS1 transgenic mice Transgenic *Caenorhabditis elegans*	2—10 μM​ (24 h) 10—20 mg/kg (3 months) 2.5–10 µM (18–36 h)	Enhanced spatial working and reference memory (higher spontaneous-alternation percentage, together with shorter acquisition paths, more platform crossings, and probe-trial time + distance in the target quadrant restored to control levels) Enhanced exploration, recognition, and spatial learning (extended locomotor activity with delayed paralysis, roughly 50% fewer head-region Aβ deposits, and a chemotaxis memory index restored toward non-transgenic levels	[64, 210, 211]
Phagocytosis + Anti-inflamation	Sesquiterpene lactone	Parthenolide		*Tanacetum parthenium* – Bai Ju	Restores microglial phagocytosis, attenuates neuro-inflammation, neuro-protection, and reduces neuronal apoptosis	Blocks Akt/MAPK cascade (ERK, JNK, p38), p65, up-regulates TRIM31, dampens NLRP3-inflammasome activation; lowers p-p65, strongly suppresses IL-6, IL-1β, TNF-α and elevates IL-10, lowers ROS, boosts SOD activity	BV2 HMC3 C57BL/6 J APP/PS1	5 µM (24 h) 2 mg/kg (7–15 days)	Improved spatial learning & memory (decreases escape latency, increases time in the target quadrant and platform crossings, but the swimming speed remains unchanged)	[159]
Antioxidant + Phagocytosis	Alkaloid	Piperine		*Piper nigrum* – Hu Jiao	Reduces oxidative stress	Reduces MDA levels, restores SOD, catalase, and glutathione (GSH), decreases IL-1β, IL-6, TNF-α, and promotes IL-4, IL-10. It also reversed microglial activation, shifting the microglia from the M1 phenotype to the M2 phenotype	C57BL/6	2.5, 5, and 10 mg/kg (15 days)	Improved cognitive performance (reduced escape latencies and increased time spent in the target quadrant)	[212]
Autophagy + anti-inflammation	Stillbenoid	Pterostilbene		*Pterocarpus marsupium*	Reduces hippocampal neuronal loss, suppresses neuro-inflammation	Strengthens Triad3A-mediated ubiquitination remains leading to lysosomal degradation of TLR4, dampening IκB-α/NF-κB signalling and lowering IL-1β & iNOS expression in microglia, directly binds MD2, disrupts MD2–TLR4 interaction, prevents TLR4 dimerization and downstream NF-κB activation, participates TLR4-mediated inflammatory response and autophagy-dependent Aβ1–42 endocytosis	Swiss-Kunming mice BV-2	10 – 40 mg/kg (24 h) 10 µM	Enhanced working and spatial learning/reference memory (alternation rate fully restored in the Y-maze; training escape latencies shortened; probe-trial crossings, time, and swim distance in the target quadrant increased; open-field locomotion unchanged, confirming cognitive-specific benefits)	[150, 151]
Autophagy + Antioxidant	Flavonoid	Silibinin		*Silybinisus labinum* L. – Shui Fei Ji	Antioxidant & neuro-protective, inhibits Aβ aggregation & preserves cognition	Activates PI3K/Akt (Ser473)/mTOR, lowering LC3-II & Beclin-1 and suppressing excessive autophagy, raises Bcl-2, cuts Bax, and restores procaspase-3, blocking mitochondrial apoptotic signaling. Scavenges ROS, reducing oxidative stress damage, and inhibits Aβ fibril formation	Primary cortical neurons from C57BL/6 mice Male C57BL/6 mice	1–10 µM 100–200 mg/kg/day (3 days)	Enhanced spatial learning and memory (reduced escape latency and increased time + distance in the target quadrant on the probe trial) Improved neurological outcome after ischemia. Enhanced neuronal survival in vitro	[213]

## Limitation

Critical data assessment reveals strengths, using in vitro and in vivo models with dose–response validation. However, limitations include species variability, cell line inconsistencies, methodological gaps, and the lack of lysosomal pH measurement. Most studies infer dysfunction from downstream markers. Publication bias and differences in compound sourcing complicate comparisons.

Most compounds modulate lysosomes indirectly via upstream pathways (e.g., TFEB/mTOR), which risks off-target effects. Early-stage disease models dominate, and compounds like α-mangostin may be ineffective in late-stage tauopathy.

Key research gaps include the lack of Phase III clinical trial data, uncertainty about lysosomal specificity, and the need for a better understanding of AD stage-specific efficacy. Dosing regimens rarely reflect chronic human exposure (3-month mouse studies with decades-long AD progression). Synergistic effects and herb-drug interactions are underexplored. Phytochemical purity varies widely, complicating bioactivity comparisons.

A deeper understanding of how phytochemicals interact with other biological pathways will be essential to assess their overall impact on AD patients' health, as their anti-inflammatory and antioxidant effects could offer additional benefits. Lastly, combining phytochemicals with existing therapies could provide a more comprehensive treatment pathway, where both approaches focus on reducing symptoms and preventing or slowing disease progression.

Developing biomarkers for the early detection of lysosomal dysfunction before neuronal damage occurs is essential to support timely intervention. Additionally, studying the genetic and environmental factors affecting lysosomal function, including gene-environment interactions and lifestyle factors, is a crucial effort that can improve the care and handling of AD.

## Conclusion

In recent years, a growing body of research has focused on the regulatory roles of phytochemicals in ameliorating lysosomal dysfunction in AD. In this review, we systematically summarized current evidence and identified five major mechanistic domains through which natural compounds exert protective effects: phagocytosis, autophagy regulation, lysosomal enzymatic activity, oxidative stress and inflammation control, and cellular energy regulation via AMPK signaling. Phytochemicals act across multiple interconnected levels of lysosomal homeostasis. For example, cannabidiol, curcumin, and spermidine promote microglial phagocytic uptake of Aβ; trehalose, chlorogenic acid, and celastrol stimulate autophagic flux via TFEB and AKT/mTOR pathways; amentoflavone, urolithin A, and chloroquine enhance lysosomal enzymatic activity and integrity; while resveratrol, crocin, and EGCG reduce oxidative stress and neuroinflammation through ROS scavenging and NF-κB inhibition. Furthermore, crocetin, fisetin, and kaempferol modulate AMPK signaling to improve cellular energy balance and lysosomal biogenesis. Multi-target compounds like methylated resveratrol, piperine, and EGCG may offer broader therapeutic potential than single-target agents.

Despite the encouraging findings, several limitations remain. The therapeutic effects of phytochemicals have shown variability across different experimental models, particularly in transgenic mouse models. The lack of robust in vivo evidence on the combinatory use of multiple phytochemicals further complicates their translational potential. Moreover, although some compounds—such as resveratrol, epigallocatechin, and tanshinone II—have been shown to cross the blood–brain barrier and exert neuroprotective effects, their clinical applicability is constrained by poor bioavailability and dose-dependent toxicity [160–162].

To address these obstacles, future research should focus on several key areas: enhancing lysosomal biogenesis, stabilizing lysosomal membrane integrity, and improving lysosomal responsiveness to cellular signals. Concurrently, the development of efficient drug delivery systems is essential to improve brain penetration and reduce systemic toxicity. Furthermore, whether these compounds can achieve adequate concentrations in human brain tissue remains to be clarified.

Overall, phytochemicals demonstrate promising potential as lysosome-targeting agents for AD therapy, offering a multi-target strategy to restore lysosomal function and slow neurodegenerative progression. A deeper understanding of their molecular targets, synergistic interactions, and pharmacokinetics will be critical to facilitate their clinical translation.

## Supplementary Information

Additional file 1.

Additional file 2.

Additional file 3.

Additional file 4.

## Data Availability

Not applicable.

## Abbreviations

AD: Alzheimer disease
AMPK: Adenosine monophosphate-activated protein kinase
APP: Amyloid precursor protein
Atg: Autophagy-related gene
ATP: Adenosine triphosphate
Aβ: Amyloid beta
BACE1: Beta-secretase
LAMP: Lysosome-associated membrane proteins
LC3: Light chain protein-3
mTOR: Mechanistic target of rapamycin
PINK1: PTEN-induced putative kinase 1
PSEN: Presenilin-
Rab7: Ras-related protein-7
RER: Rough endoplasmic reticulum
ROS: Reactive oxygen species
SIRT 1: Sirtuin-1
TFEB: Transcription factor EB
ULK: UNC-51, Like Autophagy Activating Kinase
