# Supplementary figures and images for: Potential of phytochemicals in the treatment of Alzheimer disease by modulating lysosomal dysfunction: a systematic review
Source: Chin Med. 2025 Sep 1;20:138. doi: 10.1186/s13020-025-01204-z (PMC12400566; doi:10.1186/s13020-025-01204-z)

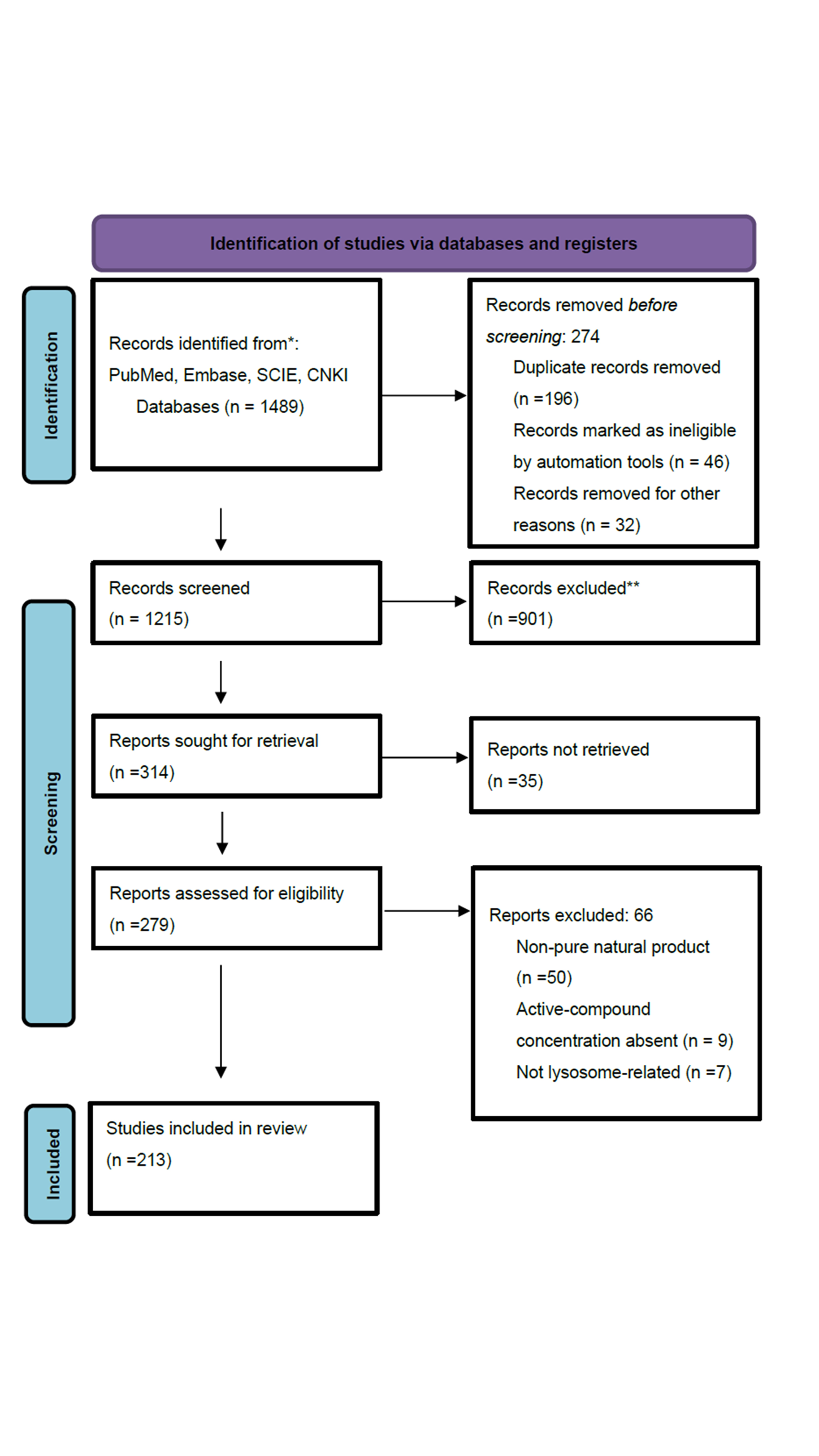

Supplement: Supplementary file 1 — Additional file 1. [file 13020_2025_1204_MOESM1_ESM.jpg]

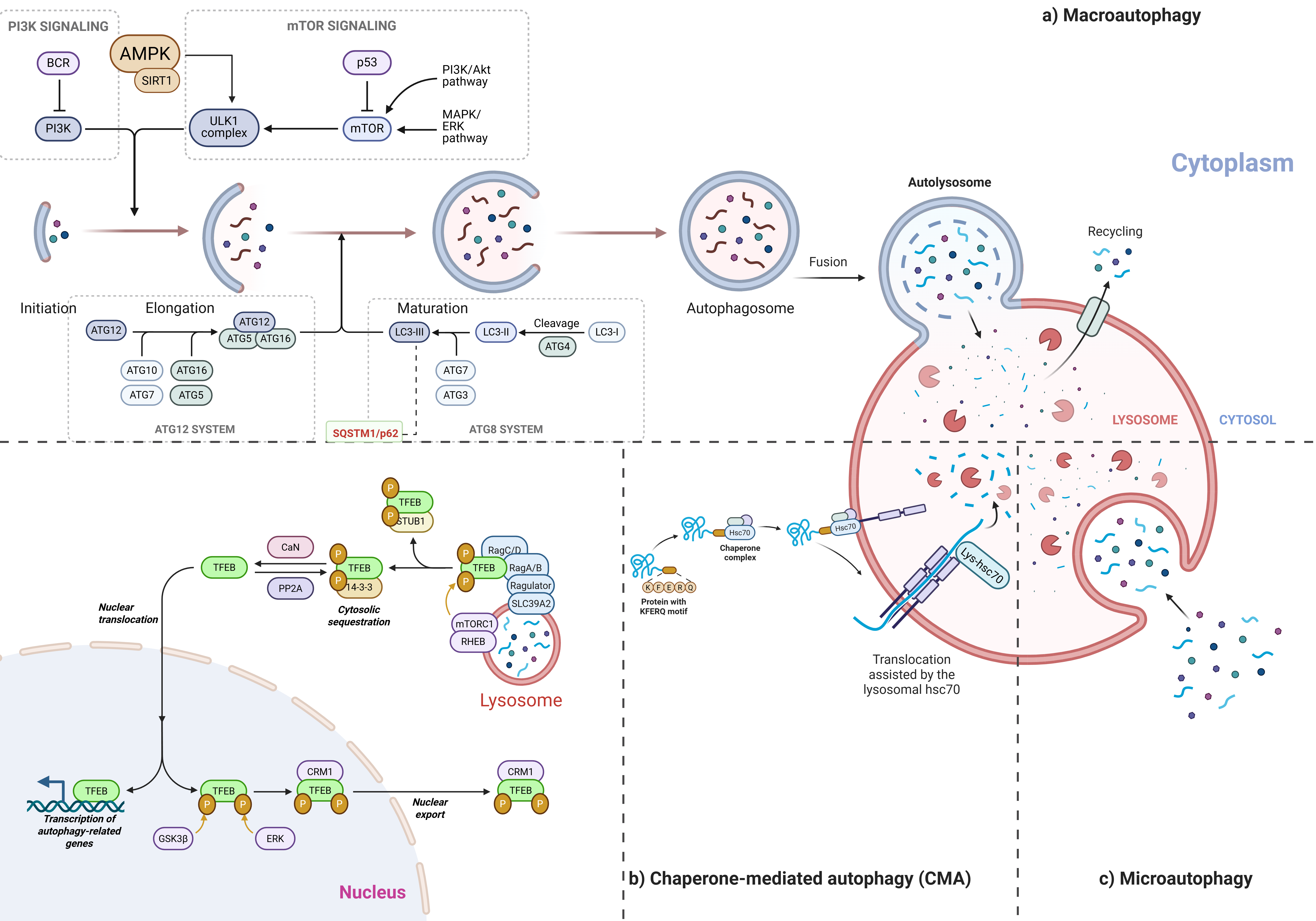

Supplement: Supplementary file 3 — Additional file 3. [file 13020_2025_1204_MOESM3_ESM.png]

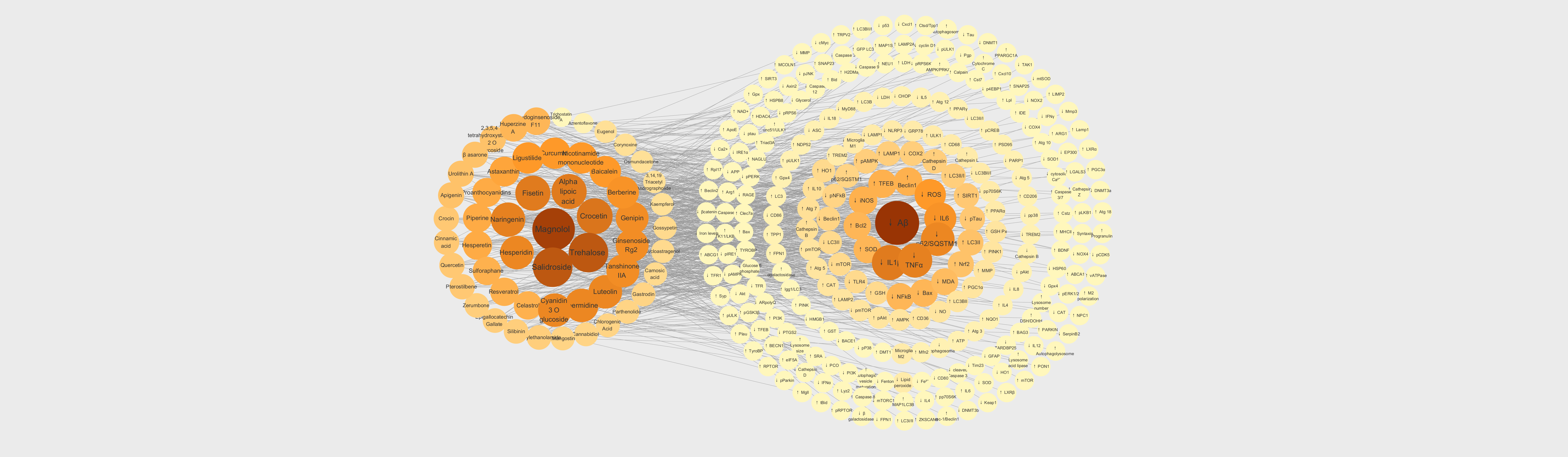

Supplement: Supplementary file 4 — Additional file 4. [file 13020_2025_1204_MOESM4_ESM.png]
